# Continuous‐Flow Microfluidic Synthesis Enhances C_2+_ Selectivity for Cu_2_O Catalysts

**DOI:** 10.1002/advs.76512

**Published:** 2026-07-14

**Authors:** Carlota Casas, Anh Tuan Ngo, João Pedro Vale, Ona Falcó, Gerard Martí, Mohamed Amazian, Júlia Mayans, Sònia Estradé, Marcos Gil‐Sepulcre, Teresa Andreu, Francesca Peiró, Tiago Sotto Mayor, Lluís Yedra, Jordi García‐Antón, Josep Puigmartí‐Luis, Xavier Sala, Roc Matheu

**Affiliations:** ^1^ Departament de Química Inorgànica i Orgànica Institut De Química Teòrica i Computacional Universitat De Barcelona Barcelona Spain; ^2^ Departament de Química Universitat Autònoma de Barcelona Cerdanyola del Vallès Spain; ^3^ Departament de Ciència de Materials i Química Física Institut de Química Teòrica i Computacional Universitat de Barcelona Barcelona Spain; ^4^ CEFT – Transport Phenomena Research Centre ALiCE – Associate Laboratory in Chemical Engineering Faculty of Engineering University of Porto Porto Portugal; ^5^ Department of Electronics and Biomedical Engineering Institute of Nanoscience and Nanotechnology Universitat de Barcelona Barcelona Spain; ^6^ Departament de Química Orgànica Universitat de València València Spain; ^7^ Institució Catalana de Recerca i Estudis Avançats Barcelona Spain

**Keywords:** CO_2_‐to‐C_2+_ electrocatalysis, copper(I) oxide nanoparticles, microfluidic technologies, reaction‐diffusion area

## Abstract

The electrochemical reduction of CO_2_ to multicarbon (C_2+_) products offers a promising pathway to replace fossil fuels in the chemical and transportation sectors. However, achieving high C_2+_ selectivity requires precisely engineered structures, which, in turn, necessitate advanced synthetic strategies and in situ characterization. Herein, we leverage microfluidic technologies to rationally design and synthesize Cu_2_O nanoparticles with tunable features under laminar flow conditions, thereby providing a previously inaccessible level of control over catalyst structure. By tuning flow parameters within the microfluidic platform, we precisely regulate the reaction‐diffusion interface, enabling fine control over nanoparticle size, morphology, and defect density. The resulting Cu_2_O nanoparticles exhibit a high defect density and intrinsic nanoporosity, two properties known to enhance C_2+_ selectivity during CO_2_ electroreduction. In contrast, Cu_2_O nanoparticles synthesized via conventional batch methods under identical stoichiometric conditions exhibit larger pore sizes, lower defect densities, and lower C_2+_ selectivity. Using in situ liquid‐phase transmission electron microscopy and operando X‐ray absorption spectroscopy, we further elucidate the evolution of both catalyst systems. Finally, we demonstrate that surface modification with polyaromatic films further promotes C_2+_ formation. This work highlights microfluidic synthesis as a powerful platform for designing advanced electrocatalysts with tunable structural features and enhanced CO_2_ conversion performance to C_2+_ products.

## Graphical abstract

Microfluidic devices are used to prepare Cu_2_O nanoparticles under laminar flow conditions, which exhibit enhanced selectivity in the CO_2_‐to‐C_2+_ reaction due to their nanoporosity and high defect concentration. Cu_2_O prepared using standard solution methods displays decreased CO_2_‐to‐C_2+_ selectivity due to the absence of similar properties. Altogether, we showcase the utility of continuous‐flow microfluidic technology in the synthesis of redox catalysts.

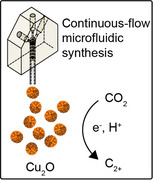

## 1 Introduction

The efficient conversion of small molecules (e.g., CO_2_, N_2_, H_2_O) into fuels or value‐added chemicals (e.g., ethylene [1, 2], NH_3_, [3, 4] H_2_ [5, 6]) requires fast, stable, and selective catalysts. Cu_2_O nanoparticles have been identified as the most promising catalysts for the CO_2_ reduction reaction to higher‐value carbon products (e.g., multicarbon products, C_2+_) due to their ability to promote C─C bond formation [7, 8]. To optimize their catalytic performance, the reactivity of Cu_2_O nanoparticles can be tuned by modifying the size, shape, surface composition, and support [9, 10]. Notably, increasing the exposure of undercoordinated defect sites enhances C_2+_ selectivity by promoting surface‐adsorbed CO coupling [11, 12, 13, 14]. Nanoporous Cu_2_O amplifies these effects by simultaneously providing abundant defect sites and enabling local CO_2_ enrichment and pH modulation, thereby further driving C_2+_ formation [15, 16, 17]. To generate a higher density of uncoordinated surface sites, several top‐down approaches (e.g., anodizing, [18, 19] oxygen plasma treatment [20]) have been developed to create nanostructured Cu surfaces exhibiting these features. However, despite their potential, many of these methods involve high‐energy processes and often yield surface morphologies with limited nanoporosity. Therefore, bottom‐up approaches that can concurrently yield undercoordinated sites and nanoporous architectures are highly desirable [15, 21].

Conventional bulk bottom‐up synthetic methods are generally ill‐suited to producing nanostructures with controlled nanoporosity and accessible coordination sites, as turbulent mixing limits control over nucleation and growth [22]. In contrast, continuous‐flow microfluidic devices have recently emerged as powerful platforms to overcome these limitations by enabling precise control of reaction‐diffusion conditions and mass transport [23, 24]. In continuous‐flow microfluidics, this level of control enables access to non‐equilibrium crystallization pathways and has been shown to direct defect formation in crystalline materials, including the generation of non‐stoichiometric structures and charge‐imbalance defects that are inaccessible under conventional bulk conditions [25]. Such control arises from the laminar‐flow regime inherent to microfluidic systems, where mixing between co‐flowing streams is governed exclusively by molecular diffusion rather than convection [26, 27]. Under these conditions, reaction‐diffusion processes and the evolution of concentration gradients can be precisely defined in space and time, providing direct control over nucleation and growth pathways and, consequently, over the resulting nanostructure. Notably, control of reaction‐diffusion conditions under laminar flow is not found in segmented microfluidic devices based on droplets, [22] nor in microfluidic setups incorporating serpentine [28] or helicoidal micromixers, [29, 30] and related geometries [31]. In those devices, rapid reagent homogenization is intentionally promoted by chaotic advection, Dean flows, or enhanced transverse mass transport, thereby preventing the well‐defined concentration gradients and reaction‐diffusion interfaces observed in continuous‐flow microfluidic devices (Figure S1) [22].

Herein, we report, for the first time, the use of continuous‐flow microfluidic devices to synthesize Cu_2_O nanoparticles with controlled morphology. By tuning reagent flows and diffusion within the microfluidic device, we demonstrate the generation of Cu_2_O with high defect concentrations and nanopores. The combination of these two properties results in enhanced C_2+_ selectivity compared with nanoparticles prepared by conventional solution methods, which exhibit larger nanopores and lower defect concentration. To advance scalability, the best Cu_2_O_fluidic_ catalyst was integrated into a flow cell. Overall, we show that the unique reaction‐diffusion environment within the continuous‐flow microfluidic device yields nanoparticles with remarkable C_2+_ selectivity, thereby providing a versatile synthetic handle for the controlled design of nanocatalysts for redox transformations.

## 2 Results and Discussion

### 2.1 Microfluidic Synthesis of Cu_2_O Nanoparticles

**FIGURE 1:**
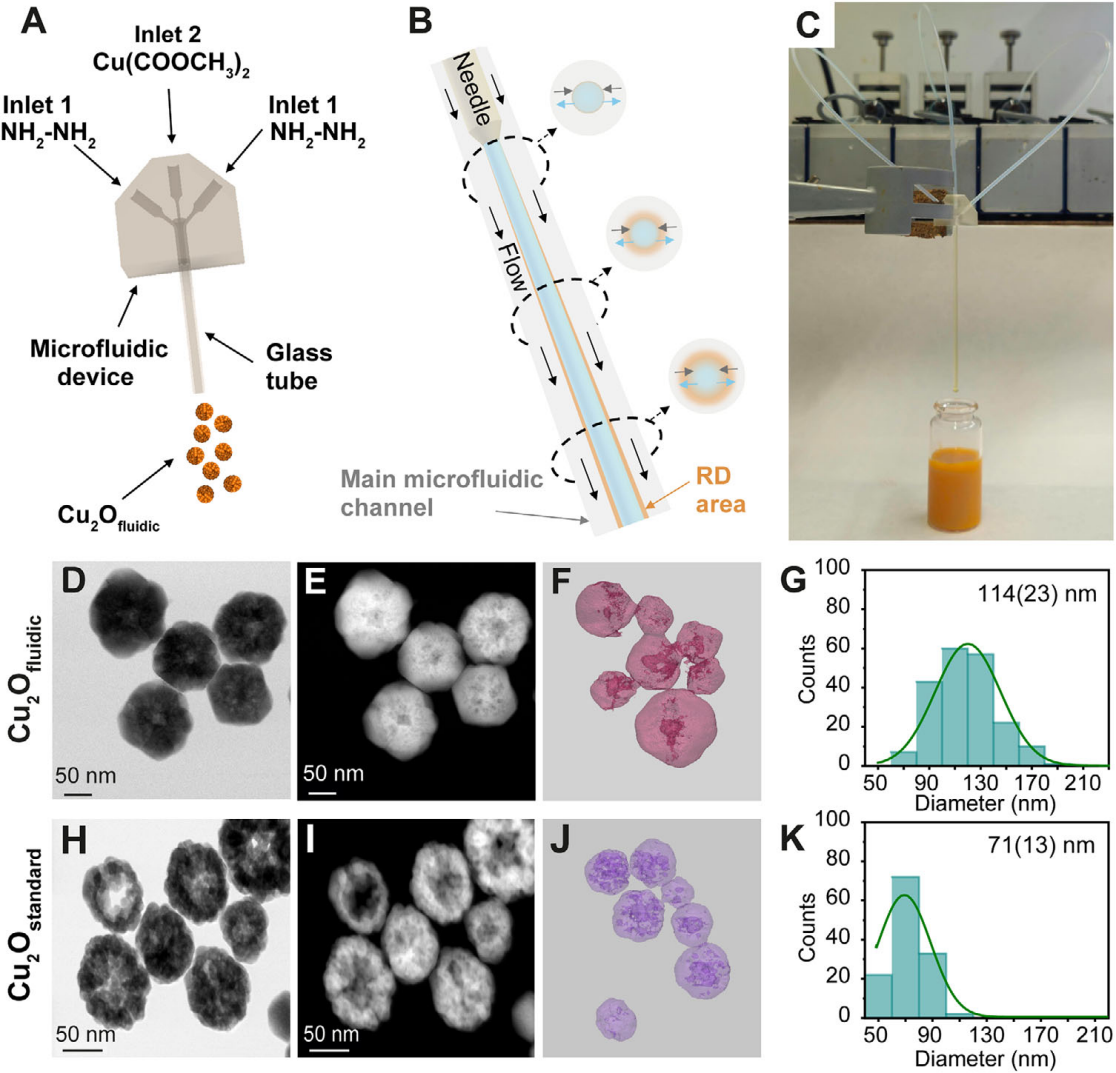
Illustration of (**A**) the microfluidic device used to synthesize Cu_2_O_fluidic_, and (**B**) the reaction‐diffusion area inside the microfluidic device. The reaction‐diffusion zone is shown in orange; the blue and grey flows are the reactants that generate the orange color (i.e., the reaction product). (**C**) Photograph of the device during Cu_2_O_fluidic_ production. (**D–G**) characterization of Cu_2_O_fluidic_, including (**D**) bright‐field scanning transmission electron microscopy (STEM) and (**E**) high‐angle annular dark‐field scanning transmission electron microscopy (HAADF‐STEM) images, (**F**) isosurface representation of the 3D reconstruction of nanoparticle shapes via HAADF‐STEM electron tomography, and (**G**) size distribution histogram. **(H–K**) Characterization of Cu_2_O_standard_, including (**H**) bright‐field STEM and (**I**) HAADF‐STEM images, (**J**) isosurface representation of the 3D reconstruction of nanoparticle shapes via HAADF‐STEM electron tomography, and (**K**) size distribution histogram.

Figure 1A–C shows the microfluidic device used to synthesize the Cu_2_O nanoparticles. It consists of three inlets connected to a linear main reaction channel. The main channel is a glass tube with an inner diameter of 1 mm and a length of 6 cm, press‐fitted into the 3D printed head (Figure S2). This setup enables a coaxial flow configuration within the main channel, in which the central stream from the central inlet (blue) is surrounded by the sheath flow (grey) from the side inlets, resulting in a concentric reaction‐diffusion zone at the liquid‐liquid interface between them (Figure 1B). As the two reagent streams move along the channel, they diffuse radially, leading to a reaction‐diffusion zone that gradually expands, as illustrated in orange in Figure 1B. This region denotes the evolving reaction‐diffusion interface at which the desired chemical reactions occur, driven by the controlled diffusion of the reagents under laminar flow conditions. The degree of diffusion within the channel can be precisely controlled by adjusting the total flow rate (TFR), defined as the sum of the flow rates from all three inlets, and the flow rate ratio (FRR), i.e., the ratio of the sheath flow rate to the central flow rate [31, 32, 33]. Here, TFR controls the residence time of the reactants within the device, and FRR determines the degree of focusing of the central flow and the location of the concentric reaction‐diffusion zone generated between the sheath and central inlet flows [34].

For the microfluidic synthesis of Cu_2_O nanoparticles (Cu_2_O_fluidic_ hereafter), we adapted a solution‐based method that uses NH_2_NH_2_ and Cu(II) acetate as reagents [35]. This particular synthetic method was chosen because it proceeds at ambient temperature and does not require gases (e.g., H_2_) or a strict O_2_‐free environment—conditions that are challenging to implement in continuous‐flow microfluidic devices. In a typical experiment, we injected an NH_2_NH_2_ solution (12.5 mM) through the side inlets (inlet 1) at a flow rate of 62.5 µL min^−1^ and a Cu(II) acetate solution (12.5 mM) through the central inlet (inlet 2) at 125 µL min^−1^, yielding a synthetic condition with an FRR of 1 and a TFR of 250 µL min^−1^. To understand how reactant concentration and Cu_2_O reaction kinetics evolve in our microfluidic device, we performed numerical simulations of flow and mass transport in these conditions, which confirmed that Cu(II) acetate and NH_2_NH_2_ solutions mix exclusively by diffusion along the device, leading to the generation of a ring‐shaped reaction‐diffusion zone where Cu_2_O is formed (Figures S34 and S35) and additional details in Section 2 in the Supporting Information). With this 3D configuration of the reaction‐diffusion zone, we observed continuous formation of Cu_2_O nanoparticles at the outlet, as evidenced by the orange color of the eluted suspension (Figure 1C). To prevent further evolution of the nanoparticles upon ejection from the chip, we explored which solvent could be used as a quencher (Figure S3). We found that nanoparticle size did not further evolve when ethanol was used as a quencher in the collecting flask. The production yield per unit time (0.127 mg min^−1^) provides the basis for scale‐up production via a parallelized microfluidic configuration [36, 37].

The nanoparticles ejected from the microfluidic device were characterized by powder X‐ray diffraction (PXRD), X‐ray absorption spectroscopy (XAS), and transmission electron microscopy (TEM). PXRD patterns of Cu_2_O_fluidic_ exhibit intense diffraction peaks, matching the expected Bragg reflections of Cu(I) oxide and discarding the presence of Cu(II) oxide (Figure S4). The identity of the formed species was further corroborated by X‐ray absorption near‐edge structure (XANES) and extended X‐ray absorption fine structure (EXAFS) analysis of Cu_2_O_fluidic_, which are fully consistent with Cu_2_O species (Figure S5). TEM images show microscopic spheres with a diameter of 114(23) nm (Figure 1D), and energy‐dispersive X‐ray spectroscopy confirmed the presence of Cu and O (Figure S6). Finally, Fourier transform indexation of high‐resolution TEM images confirmed that the structure corresponds to Cu_2_O (Figure S7).

To elucidate the effect of the microfluidic approach on nanoparticle synthesis compared with standard methods, we also prepared Cu_2_O nanoparticles using a previously reported solution method involving the slow addition of NH_2_NH_2_ to a Cu solution (Cu_2_O_standard_ hereafter) [35]. Mixing these two solutions yielded Cu_2_O, as indicated by the PXRD patterns (Figure S4), XAS (Figure S5), and indexation of the high‐resolution TEM images (Figure S8). TEM images of Cu_2_O_standard_ show spheres with a diameter of 71(13) nm (Figure 1H), ∼40 nm smaller than Cu_2_O_fluidic_ (Figure 1D). We then performed high‐angle annular dark‐field scanning transmission electron microscopy (HAADF‐STEM) images of both nanoparticles to enhance the mass contrast and probe internal density variations. HAADF‐STEM images show that Cu_2_O_standard_ are hollower (Figure 1I) than Cu_2_O_fluidic_ (Figure 1E). Using the built‐in distance‐measurement tool across 15 images per sample, we measured the average pore size of Cu_2_O_fluidic_ (16(9) nm) and Cu_2_O_standard_ (33(14) nm), indicating that Cu_2_O_standard_ has larger pores than Cu_2_O_fluidic_ (Figure S9).

Because such 2D projections can be affected by thickness variations and overlapping features along the electron‐beam direction, they do not provide unambiguous information about the internal architecture. To overcome this limitation and directly resolve the 3D morphology, we acquired a tilt series of HAADF‐STEM images of the Cu_2_O nanoparticles with electron tomography (Figure S10). This approach enables reconstruction of the full volume of the nanoparticles, allowing a reliable assessment of hollowness and pore distribution in both samples. Figure 1F,J shows the reconstructed volumes for Cu_2_O_fluidic_ (FRR = 1, TFR = 250 µL min^−1^) and Cu_2_O_standard_. The comparison of the two reconstructed volumes clearly shows that Cu_2_O_fluidic_ have fewer holes than Cu_2_O_standard_. Additionally, the two reconstructions indicate that pore connectivity differs between the two systems. Whereas most nanopores are well connected to the outside in Cu_2_O_standard_, the pores of Cu_2_O_fluidic_ appear more confined. Animations of both volume and surface representations are available as Supporting Videos S1 and S2 for Cu_2_O_fluidic_ and Cu_2_O_standard_, respectively. Finally, we performed N_2_ gas sorption isotherms at 77 K for both Cu_2_O nanoparticle types. The Brunauer–Emmett–Teller areas for both nanoparticle types are below 25 m^2^ g^–^^1^ (Figure S11), consistent with the cavities observed by TEM. Notably, the analysis cannot distinguish between the two nanoparticle types due to their low surface areas.

**FIGURE 2:**
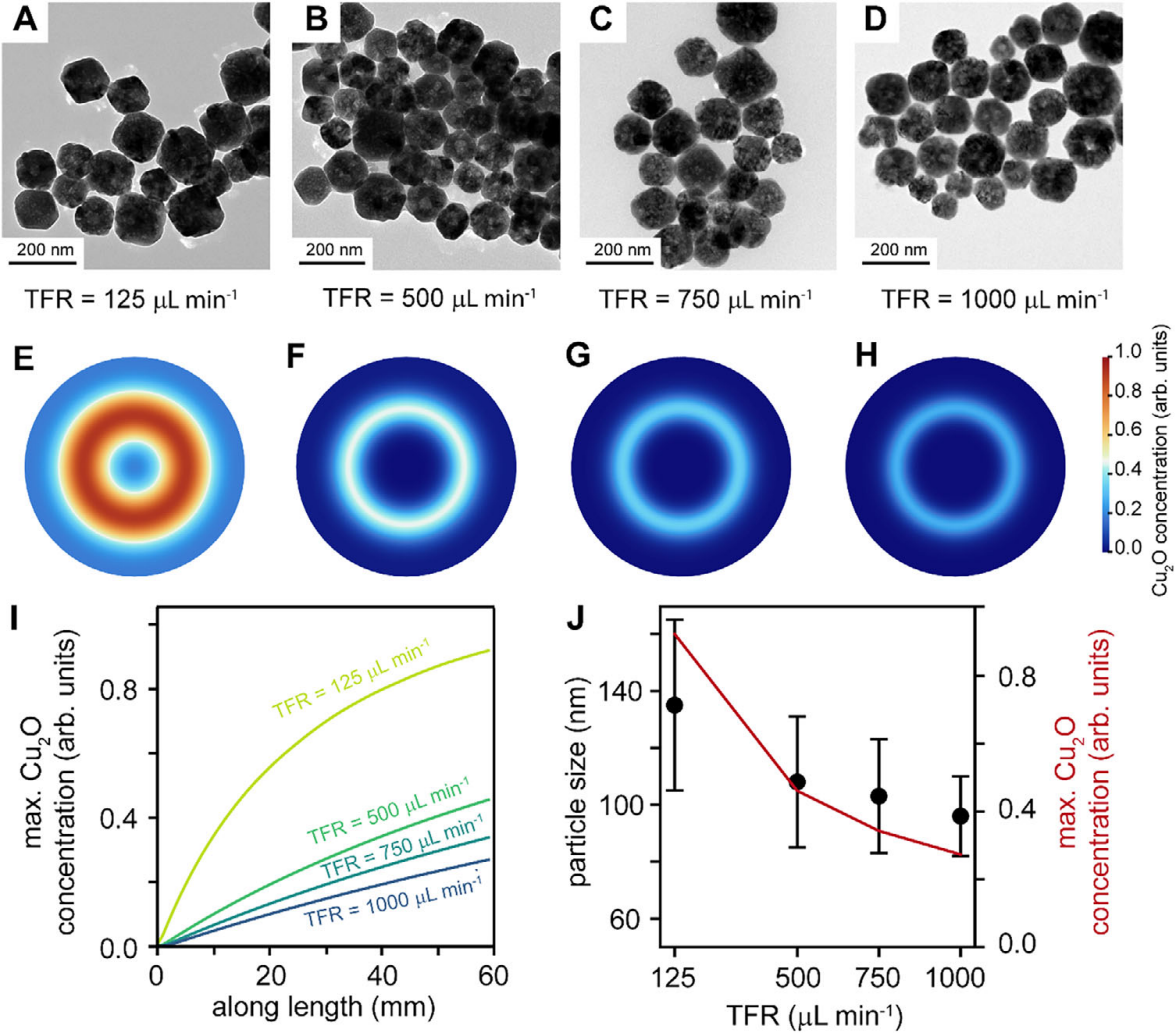
(**A–D**) TEM images for Cu_2_O_fluidic_ at the same FRR (1) but TFR of (**A**) 125, (**B**) 500, (**C**) 750, and (**D**) 1000 µL min^−1^. (**E–H**) Numerical simulation showing the Cu_2_O outlet concentration profiles for constant FRR of 1 and TFR of (**E**) 125, (**F**) 500, (**G**) 750, and (**H**) 1000 µL min^−1^. (**I**) Maximum Cu_2_O concentration along the device at different TFR. (**J**) Comparison between nanoparticle size and maximum Cu_2_O concentration. Residence times in the devices are shown in Figure S40. FRR = flow rate ratio, TFR = total flow rate.

Next, leveraging the device's capabilities to control the reaction‐diffusion zone precisely, we tuned the microfluidic synthesis parameters, namely TFR and FRR. We initially investigated the effect of TFR on the synthesis of Cu_2_O_fluidic_, and observed that increasing the TFR from 125 to 1000 µL min^−1^ for a constant FRR of 1 decreased nanoparticle size from 135(30) to 96(14) nm (Figure 2A–D). To gain insight into the relationship between nanoparticle size and reactant concentration within the device, we performed numerical simulations across the same range of TFR (Figure 2E–H; Figures S34–S39). The results indicated that increasing the TFR led to lower Cu_2_O concentrations along the device (Figure 2I) and at the outlet, due to a shorter average residence time during which reactants could diffuse and react (Figure S40). Furthermore, increasing TFR revealed a clear correlation between nanoparticle size and the maximum Cu_2_O concentration (Figure 2J), demonstrating its potential for size control. Upon TFR increase, the size distribution narrows, as indicated by a reduced standard deviation (Figure S12). This observation is supported by our numerical simulation results, which show that when the TFR was increased (e.g., to 1000 µL min^−1^), Cu_2_O occupied a more confined region of the device cross‐section (Figure 2E–H). This meant that reacting molecules and nuclei in this region experienced similar residence times and growth durations (Figure S40B), resulting in the observed narrower nanoparticle‐size distribution. Conversely, when the TFR was decreased (e.g., 125 µL min^−1^), Cu_2_O occupied most of the device cross‐section (Figure 2E). Thus, nanoparticles were generated over a wide range of residence times, including regions near the walls where residence times were very high (Figure S40B). These spatially varying growth conditions likely contribute to the larger standard deviation in nanoparticle size observed at low TFR.

To investigate the effect of flow focusing on Cu_2_O nanoparticle formation, we varied the FRR from 0.25 to 4 while keeping the TFR at 250 µL min^−1^. This change in flow focusing led to a notable difference in the nanoparticles obtained. While an FRR of 1 enabled us to obtain Cu_2_O with a diameter of 114(23) nm and uniform shape, FRRs of 0.25 and 4 yielded nanoparticles with distinct characteristics (Figure S13). When using an FRR of 0.25, we observed inhomogeneous nanoparticles with sizes and shapes ranging from ∼90 nm (some displaying sharp edges) to smaller, round nanoparticles (Figure S13A). The morphology of the nanoparticles obtained with an FRR of 0.25 is heterogeneous because Cu_2_O is generated throughout most of the device cross‐section, thus under varying compositional conditions and residence times (left plot in Figure S43C). In contrast, when using an FRR of 1, nanoparticles are generated near the interface between co‐flowing streams, forming a ring‐like structure where compositional conditions and residence times are similar (central plot in Figure S43C), thereby leading to the observed nanoparticle uniformity. Notably, when using an FRR of 4, we observe the formation of a thick layer surrounding the nanoparticles (Figure S13C). Energy‐dispersive X‐ray spectroscopy revealed a high N content in the layer (Figure S14), consistent with the increased NH_2_NH_2_ flow rate and concentration predicted by numerical simulation results during synthesis (right plot in Figure S43B). To further confirm the presence of N at the surface of Cu_2_O_fluidic_, we performed X‐ray photoelectron spectroscopy (XPS) of Cu_2_O_fluidic_ at FRR = 1 and FRR = 4 (Figure S15). In the XPS spectrum, traces of superficial N are observed for the FRR = 4 Cu_2_O_fluidic_, while no nitrogen is observed in the XPS spectra for FRR = 1 Cu_2_O_fluidic_ (Figures S16 and S17).

### 2.2 CO_2_ Reduction Electrocatalysis, Operando X‐Ray Absorption Spectroscopy, and Liquid‐Cell Transmission Electron Microscopy

**FIGURE 3:**
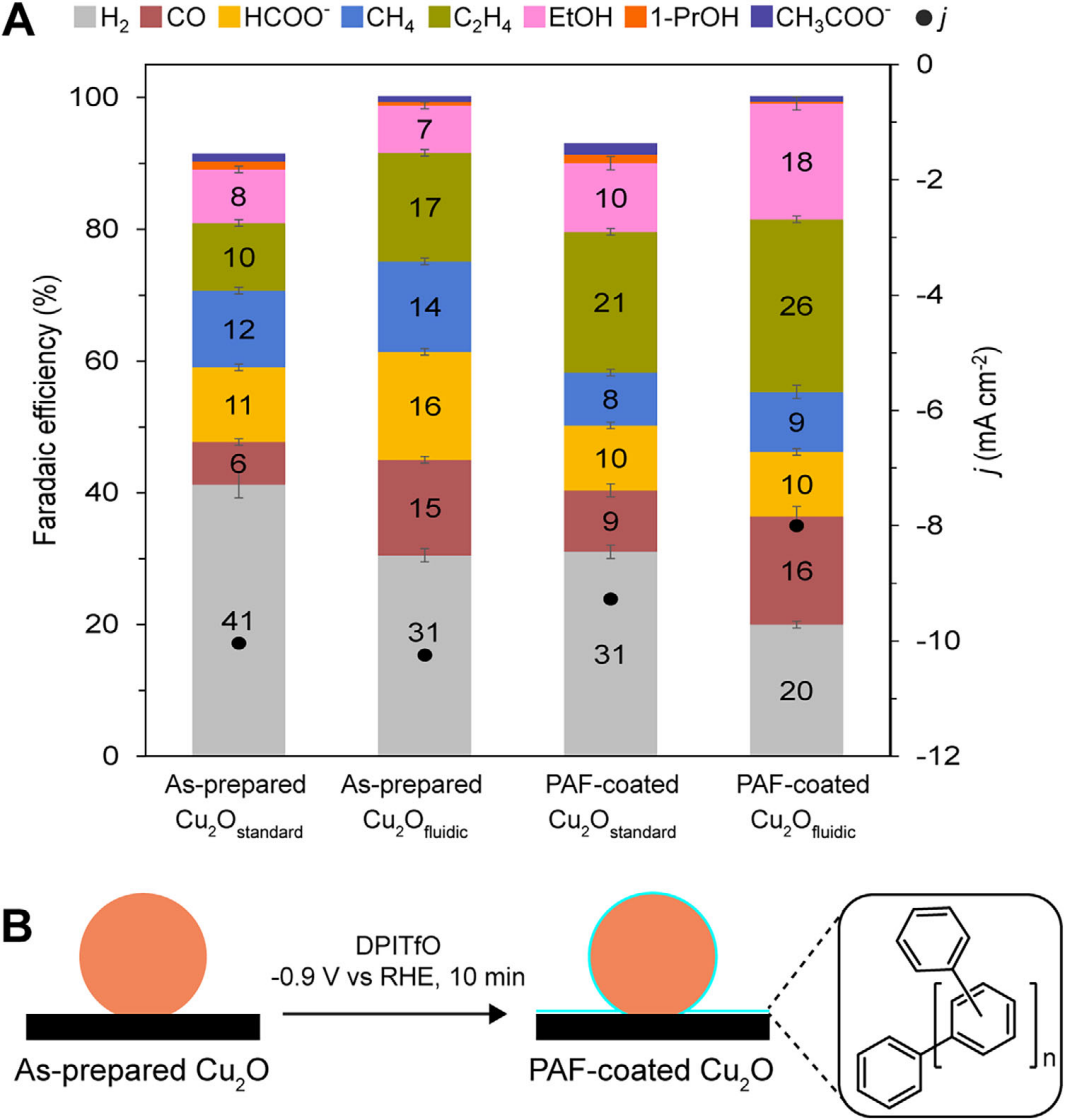
(**A**) CO_2_ electroreduction Faradaic efficiencies (bars) and current densities (black dots) for Cu_2_O_standard_, Cu_2_O_fluidic_, (FRR = 1, and TFR = 250 µL min^−1^), and PAF‐coated Cu_2_O. Cu_2_O were prepared either via standard synthesis (Cu_2_O_standard_) or microfluidic synthesis (Cu_2_O_fluidic_, FRR = 1, and TFR = 250 µL min^−1^). A potential of –1.25 V vs RHE was applied for 1 h. (**B**) Synthetic scheme for the polyaromatic films (PAF) coating process of Cu_2_O under reductive electrochemical conditions in a 0.1 M KHCO_3_ solution containing 10 mM DPITfO aqueous solution. DPITfO = diphenyl iodonium triflate.

Having demonstrated that our microfluidic approach enables the generation of Cu_2_O nanoparticles with tailored morphology, we first characterized their electrocatalytic properties for the CO_2_ reduction reaction in a two‐compartment gas‐tight H‐cell filled with KHCO_3_ solution and purged with CO_2_. The working electrode consisted of drop‐casted Cu_2_O nanoparticles on a carbon paper electrode. We initially evaluated nanoparticles prepared at an FRR of 1 and a TFR of 250 µL min^−1^ (Cu_2_O_fluidic_). Chronoamperometry was performed at −1.05, −1.25, and −1.40 V vs RHE for 1 h, and CO_2_ reduction products were quantified by gas chromatography (Figure S18A) and ^1^H NMR spectroscopy (Figure S18B) to calculate Faradaic efficiencies (Figure S19A). Among the tested potentials, −1.25 V vs RHE was identified as the optimal operating condition, yielding the highest selectivity for C_2+_ products (25%) and the lowest selectivity for H_2_ (31%) (Figure 3A and Figure S19A, and Table S1). In comparison, Cu_2_O_standard_ shows lower C_2+_ selectivity (20%) and higher H_2_ selectivity (41%) under identical conditions (Figure 3A and Table S1). These results confirm that microfluidic synthesis yields Cu_2_O nanoparticles with intrinsically enhanced C_2+_ selectivity.

Based on this initial screening, −1.25 V vs RHE was selected for subsequent studies to evaluate the influence of microfluidic flow conditions on catalytic performance. Using this optimized potential, Cu_2_O_fluidic_ samples prepared at different FRR values (0.25, 1, and 4) were then compared, with FRR = 1 serving as the reference condition identified in the initial tests (Figure S19B). Relative to this benchmark, both lower and higher FRR values result in decreased C_2+_ selectivity, confirming that the optimum observed at FRR = 1 reflects a true performance maximum, with less H_2_ production. Nanoparticles with increased TFR have also been tested at –1.25 V vs RHE to evaluate their performance in CO_2_ reduction electrocatalysis. At increased TFR (500, 1000 µL min^−1^), a higher selectivity for H_2_ is observed (Figure S20). We attribute the enhanced H_2_ selectivity at high TFR to the smaller nanoparticle diameter, as small nanoparticles are known to favor H_2_ reduction due to poorer stabilization of CO_2_ intermediates [38]. Selectivity of Cu_2_O_fluidic_ prepared at TFR = 125 µL min^−1^ is comparable to selectivity of Cu_2_O_fluidic_ at TFR = 250 µL min^−1^, as their structures are similar. According to the characterization of all the studied systems (Section 2.1 above and Figures S40–S43), the superior performance of the FRR = 1 and TFR = 250 µL min^−1^ sample is attributed to an optimal balance between nanopore formation, size, and structural integrity, as well as the absence of a superficial nitrogen‐containing layer. These results highlight reaction‐diffusion control in microfluidics as a key parameter for tuning catalyst structure and performance.

The superior performance of Cu_2_O_fluidic_ vs Cu_2_O_standard_ can be rationalized by a synergistic interplay between nanoconfinement and defect engineering. First, nanopores act as local reaction chambers that trap CO_2_ and stabilize surface‐adsorbed CO intermediates, thereby increasing their local concentration and residence time. This confinement effect promotes CO‐CO coupling, which is the key rate‐determining step toward C_2+_ formation. Notably, the narrower pore architecture in Cu_2_O_fluidic_ further intensifies this effect by strengthening local mass‐transport limitations and enhancing intermediate accumulation [15]. Second, the reaction‐diffusion conditions inherent to microfluidic synthesis promote the formation of a high density of uncoordinated defect sites. These sites are known to bind CO intermediates more strongly and lower the barrier for C−C coupling [11, 12, 31]. The combination of defect‐rich surfaces and nanoconfined environments therefore creates a favorable catalytic landscape that drives multicarbon formation over hydrogen evolution.

To validate these structural differences, we employed Pb underpotential deposition (Pb‐UPD) to probe the surface structure of both catalysts under negative bias. Pb‐UPD is highly sensitive to the atomic arrangement of Cu surfaces due to structure‐dependent adsorption of Pb. Pb‐UPD curves show pronounced differences between reduced Cu_2_O_fluidic_ and reduced Cu_2_O_standard_ (Figure S21 and Table S2), confirming distinct surface structures and defect distributions. Cu_2_O_standard_ shows features associated with Cu(310) and Cu(111) facets, while Cu_2_O_fluidic_ is dominated by Cu(110) signatures and a higher density of defect‐related contributions. This facet distribution in Cu_2_O_fluidic_ is consistent with a surface enriched in low‐coordination sites, which are known to facilitate CO coupling and enhance C_2+_ formation [11, 12].

To complement Pb‐UPD measurements, we have elucidated the electrochemically active surface area (ECSA) and roughness factor for Cu_2_O_fluidic_ and Cu_2_O_standard_ (Figure S22 and Table S3). Due to its reduced porosity, Cu_2_O_fluidic_ exhibits a lower roughness factor (0.19) than Cu_2_O_standard_ (0.63). The ECSA‐normalized current densities of Cu_2_O_fluidic_ (–53(11) mA cm^−2^) are much larger than the Cu_2_O_standard_ one (–16(3) mA cm^−2^) (Table S1). The difference cannot be attributed to particle size, as Cu_2_O_fluidic_ has larger nanoparticles, confirming the presence of highly active sites for the CO_2_ reduction reaction observed by Pb‐UPD.

**FIGURE 4:**
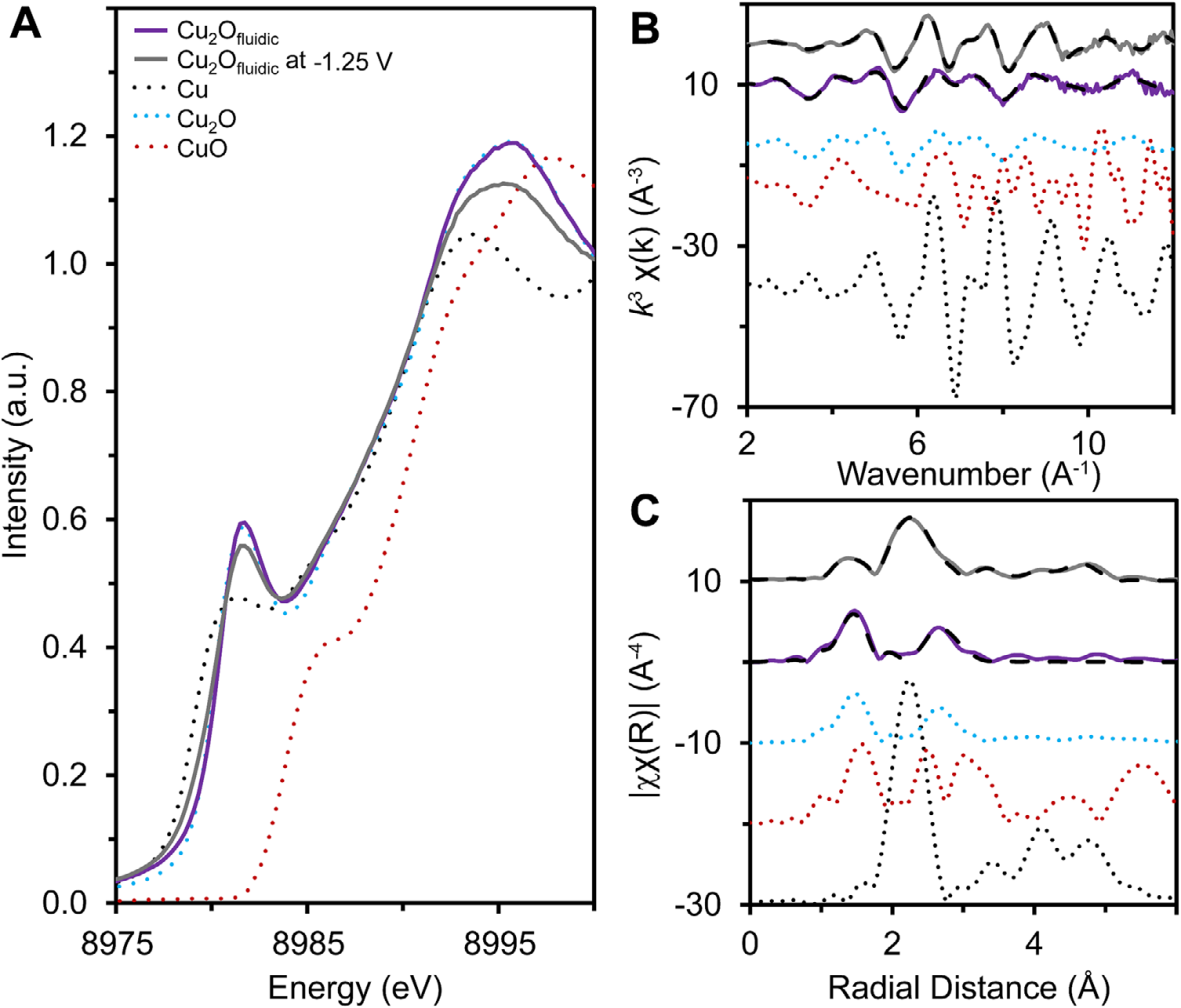
(**A**) Normalized Cu K‐edge X‐ray absorption near‐edge structure (XANES) spectra of Cu_2_O_fluidic_ before and during CO_2_ reduction catalysis (solid lines), applying at –1.25 V vs RHE in 0.1 M KHCO_3_ buffer saturated with CO_2_, along with Cu(0), Cu_2_O, and CuO references (dotted lines). (**B**) *k* ^3^‐weighted and (**C**) Fourier transform of *k* ^3^‐weighted Cu Extended X‐ray absorption fine structure (EXAFS) spectra of Cu_2_O_fluidic_ before and during CO_2_ reduction catalysis under the same conditions. Experimental data are shown as solid and dotted lines, whereas the fitted curves are displayed as dashed lines. Experimental spectra were fitted for over a *k*‐range of 2–12 Å^−1^.

To elucidate the key reactive species of Cu_2_O_fluidic_ and Cu_2_O_standard_ under catalytic conditions, we used operando X‐ray absorption spectroscopy (XAS) [39, 40]. Operando XAS allows monitoring of the evolution of the Cu K‐edge features by applying a constant potential (Figure 4 and Figures S23 and S24). The XANES spectra recorded in the absence of applied potential display edge positions consistent with Cu_2_O, overlapping with the reference spectrum and further supported by EXAFS analysis (Figure S5). The corresponding R‐space spectra for the initial Cu_2_O_fluidic_ and Cu_2_O_standard_, were fitted using a simple structural model based on Cu_2_O (see Figure 4, Figure S23, and Table S4). These are dominated by Cu–O scattering contributions at ca. 1.84 Å and by a second intense feature centered at ca. 3.02 Å, corresponding to the second (Cu–Cu) coordination shells, characteristic of Cu_2_O (Table S4). Upon applying a cathodic potential in the CO_2_ reduction region (–1.25 V vs RHE), both samples exhibit a Cu K‐edge shift to lower energies, consistent with Cu(0) formation. Edge fitting of the Cu K‐edge XANES features (Figure S24) reveals a lower reduction degree for Cu_2_O_fluidic_ (34%) than Cu_2_O_standard_ (45%), indicating distinct operando evolution and highlighting the ability of microfluidic synthesis to tune accessible oxidation states under catalytic conditions. The EXAFS analysis further corroborates the trends observed by XANES, showing that under catalytic conditions the Cu–O scattering contribution at ca. 1.84 Å decreases in intensity, accompanied by the emergence of a feature at ca. 2.56 Å, characteristic of first‐shell Cu–Cu distances in metallic Cu. Additionally, scattering contributions at greater radial distances become evident, consistent with well‐defined second‐ and third‐coordination shells in metallic Cu.

We next evaluated catalyst evolution under reaction conditions using liquid‐cell TEM. Under an applied potential of ∼–1.25 V vs RHE, Cu_2_O_fluidic_ undergoes significant restructuring, shrinking, and transforming into Cu nanoparticles in the low‐nm range (Figures S25 and S26). Electron energy‐loss spectroscopy confirms that these newly formed nanoparticles are predominantly metallic Cu (Figure S26). A similar transformation is observed for Cu_2_O_standard_ (Figure S27), consistent with the well‐known electrochemical dissolution and redeposition of Cu‐based species under CO_2_ reduction reaction conditions [41, 42, 43, 44, 45, 46].

To elucidate the catalytic role of these dynamically formed Cu species, we performed sequential chronoamperometric measurements in an H‐cell. Upon applying –1.25 V vs RHE to Cu_2_O_fluidic_, a pronounced time‐dependent evolution in product distribution was observed. The Faradaic efficiency for carbon products gradually decreases, while H_2_ production increases substantially (from 30% at 1 h to 55% at 2 h), reaching a steady‐state value of ∼54% H_2_ at 3 h (Figure S28). This shift indicates that a loss of C_2+_ selectivity accompanies the progressive restructuring of the catalyst toward small, redeposited Cu nanoparticles. These observations suggest that the highly selective catalyst state is associated with the early reconstructed structure derived from Cu_2_O_fluidic_. In contrast, continued dissolution and redeposition lead to Cu species that increasingly favor H_2_ evolution. The results, therefore, highlight the importance of controlling catalyst reconstruction through precursor design.

The operando XAS and liquid‐cell TEM experiments demonstrate that both Cu_2_O_fluidic_ and Cu_2_O_standard_ undergo significant reduction and restructuring under cathodic conditions, ultimately generating Cu‐rich catalysts. Therefore, the enhanced C_2+_ selectivity observed for Cu_2_O_fluidic_ cannot be attributed to the preservation of a static Cu_2_O structure during catalysis. Instead, these results suggest that the structural characteristics introduced during microfluidic synthesis influence the reconstruction pathway and the nature of the active Cu species formed under reaction conditions.

Importantly, the reconstructed catalysts are not identical. Operando XAS reveals different degrees of reduction for Cu_2_O_fluidic_ and Cu_2_O_standard_, while Pb‐UPD measurements performed after activation reveal distinct surface structures and defect distributions. These observations indicate that the catalyst retains a structural memory of the precursor despite extensive reconstruction. We therefore attribute the superior CO_2_ reduction catalysis performance of Cu_2_O_fluidic_ to precursor‐directed reconstruction, in which the higher defect density and confined nanoporosity generated by microfluidic synthesis promote the formation of Cu surfaces enriched in low‐coordination sites, which are favorable for C─C coupling.

Finally, to modulate the interfacial reactivity, we engineered the catalyst‐electrolyte interface using polyaromatic films (PAF). According to recent reports, hydrophobic PAF coatings may facilitate interfacial CO_2_ transport while suppressing the diffusion of H_2_O and H^+^ to the electrode surface [46, 47, 48, 49, 50, 51]. In particular, we adapted a procedure developed by Peters, Agapie, and coworkers based on the electroreduction of diphenyl iodonium triflate (DPITfO) under negative bias (Figure 3B) [52]. The PAF‐coated Cu_2_O_fluidic_ electrode exhibits a further increase in C_2+_ selectivity up to 45%, with a pronounced selectivity for ethylene (26(3)%) and ethanol (18(2)%). PAF‐coated Cu_2_O_standard_ also shows enhanced C_2+_ formation, reaching an ethylene selectivity of 21(3)% and ethanol selectivity of 10(2)% (Figure 3A). ECSA‐normalized current densities reveal that PAF‐coated Cu_2_O_fluidic_ (−124(16) mA cm^−2^) is much more active than PAF‐coated Cu_2_O_standard_ (–36(3) mA cm^−2^), indicating that the structural factors driving catalysis (i.e., nanoporosity, high concentration of defects) are maintained with PAF‐coating. Further, we analyzed the hydrophobicity of the catalysts, revealing that Cu_2_O_fluidic_ exhibits greater hydrophobicity than Cu_2_O_standard_, which likely explains its lower affinity for H_2_ evolution (Figure S29 and Table S5). The high selectivity for C_2+_ is likely due to structural factors and hydrophobicity. Overall, the enhancement of the CO_2_‐to‐C_2+_ selectivity is comparable (enhancement of 20%) to that observed in other Cu‐based catalysts modified with molecular and polymeric coatings (enhancement of 5%–50%, Table S6). These results reinforce the importance of controlling interfacial mass transport and local CO availability for C─C coupling. The hydrophobic PAF layer preferentially enhances CO_2_ diffusion while limiting H^+^/H_2_O accessibility, thereby diminishing H_2_ evolution and promoting the formation of multicarbon products [47, 48].

To evaluate the suitability of our best‐performing catalysts at practical current densities, we integrated Cu_2_O_fluidic_ with PAF in a flow cell CO_2_ reduction setup (Figure S30). We evaluated the performance of the modified gas‐diffusion layer under comparable H‐cell conditions (−1.25 V vs RHE, 0.5 M KHCO_3_) and measured the evolution of products over 3 h. The current density of Cu_2_O_fluidic_ catalysts in the flow cell is maintained at ∼ 90 mA cm^−2^ for 3 h with ∼ 40% Faradaic efficiency for C_2+_ (Figure S30). Despite differences in electrocatalytic conditions and coating, the C_2+_ selectivity of PAF‐coated Cu_2_O_fluidic_ compares well with that of other Cu_2_O prepared by mixing‐dominated microfluidic devices (32%, Table S6).

## 3 Conclusion

This work demonstrates that continuous‐flow microfluidic devices provide a precise and versatile platform for engineering Cu_2_O nanoparticles with enhanced catalytic performance. By carefully controlling reaction‐diffusion conditions under laminar flow, we can tailor nanoparticle features, such as defect density and nanoporosity, that are otherwise difficult to modulate with conventional batch synthesis methods. The Cu_2_O nanoparticles produced via microfluidic synthesis exhibit a higher defect density and more confined porosity, features that influence catalyst evolution under CO_2_ reduction conditions and promote the formation of reconstructed Cu catalysts with enhanced C_2+_ selectivity. The Cu_2_O nanoparticles produced via microfluidic synthesis exhibit a higher defect density and more confined porosity, which directly contribute to improved catalytic performance. When further modified with polyaromatic films, these materials achieve up to 45% selectivity toward C_2+_ products in CO_2_ electroreduction. In contrast, nanoparticles synthesized via standard batch methods exhibit lower defect densities, larger pore sizes, and reduced C_2+_ selectivity. In addition, in situ liquid‐phase TEM and operando XAS provide valuable insight into the evolution of nanoparticles under reaction conditions, helping to link catalyst structure with performance. The incorporation of electropolymerized polyaromatic films further enhances selectivity, underscoring the importance of surface‐modification strategies. Overall, this study establishes microfluidic synthesis as a powerful, controllable approach for designing high‐performance nanocatalysts, offering new opportunities to advance CO_2_ electroreduction toward multicarbon products.

## 4 Methods

### 4.1 General Considerations

Hydrazine monohydrate (N_2_H_4_ 64%–65%; reagent grade 98%), potassium hydrogen carbonate (KHCO_3_, reagent grade 99.7%), dimethyl sulfoxide (DMSO, ≥ 99.9%), lead perchlorate hydrate (Pb(ClO_4_)_2_, 98%), potassium perchlorate (KClO_4_, ACS reagent ≥ 99%), sodium chloride (NaCl, ≥ 99.9%) and deuterium oxide (D_2_O, 99.9%) were purchased from Sigma Aldrich. Copper acetate hydrate (Cu(CH_3_COO)_2_·H_2_O, 65%) was purchased from J.T. Chemicals. Perchloric acid (HClO_4_, 60%) was purchased from Panreac. All solutions were prepared with ultrapure water (17–19 MΩ·cm) from a Milli‐Q instrument. Tetrahydrofuran (THF) for suspensions was dried over sodium/benzophenone. Carbon paper (Sigracet 39 AA) and gas diffusion layer (GDL, Quintech) were purchased from FuelCell store. All carbon paper pieces were washed by sonication in isopropanol for 15 min, in hexane for 15 more minutes, and dried thoroughly before use. Gas mixtures containing H_2_, CO, CH_4,_ and C_2_H_4_ for gas chromatography calibration were purchased from Linde. Deuterium oxide for NMR was purchased from Eurisotop.

### 4.2 Microfluidic‐Based Synthesis of Cu_2_O_fluidic_

#### 4.2.1 General Methodology

The microfluidic‐based Cu_2_O nanoparticles were prepared using a modified version of a previously described method [35] An aqueous solution (20 mL) containing Cu(CH_3_COO)_2_·H_2_O (0.05 g, 0.25 mmol) was prepared and connected to the central channel of the coaxial flow‐focusing device (Figure 1) using a 10 mL Fisher plastic syringe. A second aqueous solution was prepared containing hydrazine hydrate (18.66 µL, 0.25 mmol) and connected to the lateral channels using 10 mL Fisher plastic syringes. The injection of the solutions into each inlet was controlled by high‐precision computer‐controlled syringe pumps (CETONI Low Pressure Syringe Pump neMESYS 290N) using software (Nemesys UserInterface). The orange suspension was collected continuously from the outlet into a vial immersed in ethanol. The nanoparticles were isolated by centrifugation (Eppendorf Centrifuge 5810) from the suspension and washed three times with Milli‐Q water and three times with ethanol. The nanoparticles were dried under vacuum overnight and stored in a N_2_‐filled glovebox.

#### 4.2.2 Preparation of Cu_2_O_fluidic_ With TFR = 125 µL min^−1^ and FRR = 1

A 62.5 µL min^−1^ flow rate for the central and 31.25 µL min^−1^ flow rate for the lateral inlets. The orange suspension was continuously collected from the outlet into a vial containing ethanol, yielding a rate of 0.188 mg min^−1^.

#### 4.2.3 Preparation of Cu_2_O_fluidic_ With TFR = 250 µL min^−1^ and FRR = 1

A 125  µL min^−1^ flow rate for the central and 62.5 µL min^−1^ flow rate for the lateral inlets. The orange suspension was continuously collected from the outlet into a vial immersed in ethanol, yielding a rate of 0.127 mg min^−1^.

#### 4.2.4 Preparation of Cu_2_O_fluidic_ With TFR = 500 µL min^−1^ and FRR = 1

A 250  µL min^−1^ flow rate for the central and 125 µL min^−1^ flow rate for the lateral inlets. The orange suspension was continuously collected from the outlet into a vial containing ethanol, yielding a rate of 0.061 mg min^−1^.

#### 4.2.5 Preparation of Cu_2_O_fluidic_ With TFR = 750 µL min^−1^ and FRR = 1

A 375  µL min^−1^ flow rate for the central and 187.5 µL min^−1^ flow rate for the lateral inlets. The orange suspension was continuously collected from the outlet into a vial containing ethanol, yielding a rate of 0.033 mg min^−1^.

#### 4.2.6 Preparation of Cu_2_O_fluidic_ With TFR = 1000 µL min^−1^ and FRR = 1

A 500  µL min^−1^ flow rate for the central and 250 µL min^−1^ flow rate for the lateral inlets. The orange suspension was continuously collected from the outlet into a vial containing ethanol, yielding a rate of 0.017 mg min^−1^.

#### 4.2.7 Preparation of Cu_2_O_fluidic_ with TFR = 250 µL min^−1^ and FRR = 0.25

A 200  µL min^−1^ flow rate for the central and 25 µL min^−1^ flow rate for the lateral inlets. The orange suspension was continuously collected from the outlet into a vial immersed in ethanol, yielding a rate of 0.041 mg min^−1^.

#### 4.2.8 Preparation of Cu_2_O_fluidic_ With TFR = 250 µL min^−1^ and FRR = 4

A 50  µL min^−1^ flow rate for the central and 100 µL min^−1^ flow rate for the lateral inlets. The orange suspension was continuously collected from the outlet into a vial immersed in ethanol, yielding a rate of 0.211 mg min^−1^.

### 4.3 Synthesis Cu_2_O_standard_

The nanoparticles were prepared by modifying a previously described method [35]. We dissolved Cu(CH_3_COO)_2_·H_2_O (0.25 g, 1.25 mmol) in 100 mL of milli‐Q water. After stirring for 30 min, hydrazine hydrate (93.28 µL, 1.25 mmol) was added dropwise. The solution underwent a visible colour change from pale blue to bright orange. The mixture was left stirring at room temperature for 30 min. The nanoparticles were isolated by centrifuging the suspension (Eppendorf Centrifuge 5810) and washing three times with Milli‐Q water and three times with ethanol. The nanoparticles were dried under vacuum overnight and stored in a N_2_‐filled glovebox.

### 4.4 Microfluidic Device Preparation

The microfluidic device was designed using computer‐aided design (CAD) software (Autodesk Fusion, USA) and fabricated via 3D printing using a ProFluidics 285D printer (CADworks3D, Canada). Figure S2 shows the design. The system comprises two primary components: a 3D‐printed device head and a glass capillary. The head features three fluid inlets and a single outlet housing a concentric central needle. A glass tube (length: 6 cm; inner diameter: 1 mm) was press‐fitted into the outlet to serve as the main microfluidic channel.

### 4.5 Powder X‐ray diffraction

Powder X‐ray diffraction (PXRD) measurements were performed at the Centres Científics i Tecnològics de la Universitat de Barcelona (CCiTUB) under ambient conditions on a *PANalytical Empyrean alpha1* powder diffractometer equipped with a Cu anode. The instrument was operated in a Bragg‐Brentano geometry with a step size of 0.017° (*2θ*) and a measuring time of 60 s. Start and final *2θ* angles were 4–100°. Simulated powder patterns were calculated using Mercury software and the crystallographic information files (CIFs) from single‐crystal X‐ray structures.

### 4.6 Transmission Electron Microscopy (TEM)

#### 4.6.1 Instrumentation

Imaging and analytical (scanning) transmission electron microscopy‐(S)TEM was performed at CCiTUB by means of a JEOL JEM 2010F working at 200 kV acceleration voltage, equipped with a field emission gun, a GATAN image filter (GIF) for electron energy loss spectroscopy (EELS), and a STEM unit with a high‐angle annular detector. Electron tomography was performed using a JEOL JEM ARM200 (NEOARM) unit with a cold field‐emission gun, equipped with a GIF Continuum K3 for EELS and an electrostatic dose modulator, operating at 200 kV.

#### 4.6.2 Sample Preparation

All samples for TEM, EELS, EDX, and electronic tomography analyses were prepared by slow evaporation of a drop of Cu_2_O suspension in ethanol, followed by deposition onto a holey carbon‐covered gold grid.

#### 4.6.3 Data Acquisition and Treatment

Electron tomography was performed in high‐angle annular dark‐field scanning transmission electron microscopy (HAADF‐STEM) mode to avoid non‐linear diffraction contrast contributions to the images. Tilt series ranged from ‐69° to +60°, with images acquired every 1°. Alignment of the images and reconstruction was carried out with *Temography Composer* software using 20 iterations of SIRT algorithm [53]. All shown visualizations of the reconstructed volume were obtained from the Tomviz software. TEM micrographs were analyzed in *ImageJ* for particle size assessment. In each sample, at least 250 nanoparticles from 4 different grid regions were measured to ensure representative results across all Cu‐based materials. Pore size measurements were performed using Gatan Digital Micrograph software, with the built‐in distance‐measurement tool used to determine visible pore diameters from HAADF images manually. In total, 147 pores were measured for each sample (294 pores in total), and statistical analysis was subsequently performed using the extracted values.

### 4.7 Liquid‐Cell TEM

#### 4.7.1 Instrumentation

The in situ TEM imaging was performed at CCiTUB using a JEOL JEM‐ ARM200 (NEOARM) operating at 200 kV. Electron beam conditions were selected to optimize imaging while maintaining a low overall electron dose controlled via an electrostatic beam modulator. At these dose rates, we did not observe obvious effects of the electron beam; dynamic changes were observed only during the application of the potential. The observations were conducted in STEM mode.

The observations were performed in a liquid‐biasing DENSsolutions Stream holder and the corresponding chip with tree Pt electrodes and silicon nitride membrane regions as electrotransparent windows encapsulating the sample (no liquid flow was applied). The LC‐TEM holder was leak tested in a Pfeifer vacumm HiCUBE pumping station prior to the introduction in the TEM. The electrochemical experiments were conducted using a PalmSens4 potentiostat.

#### 4.7.2 Sample Preparation

Samples were prepared by slow evaporation of a drop of Cu_2_O suspended in ethanol, followed by deposition onto the bottom chip, which contains the electrodes. Once the ethanol evaporated, a drop of 0.1 M KHCO_3_ electrolyte saturated with CO_2_ was drop‐cast onto the stationary cell chip. The bottom chip was then covered with the top chip containing the window, both parts aligned, and the cell was sealed.

#### 4.7.3 Data Acquisition and Treatment

Linear sweep voltammetry was performed at a scan rate of 25 mV s^−1^, ranging from 0 to –2500 mV vs Pt pseudo‐reference electrode. Chronoamperometry (CA) was performed by applying the selected potential for 300 s. We identified the potential (∼–1.25 V vs RHE) based on the signal‐to‐noise ratio of the cyclic voltammetry current from the H‐cell. STEM images were taken before and during each electrochemical experiment. During the experiments, STEM images were taken at ∼1 frame s^−1^.

### 4.8 Electrode Preparation

We drop‐casted 0.5 µL of Cu_2_O_fluidic_ and Cu_2_O_standard_ suspensions (0.5 mg mL^−1^ in THF) onto each side of carbon paper pieces (1.5 × 0.5 cm). Conductive copper tape was attached to each carbon paper‐based electrode, and Teflon tape was used to adjust the exposed area. We used carbon paper pieces as working electrodes during the electroreduction of CO_2_.

The polyaromatic film (PAF) coatings were generated in a one compartment cell containing an aqueous CO_2_‐saturated (0.1 M KHCO_3_) 10 mM diphenyliodonium triflate (DPITfO) solution. To generate the PAFs, –0.90 V vs RHE were applied for 10 min. After that, the electrode was removed from the gas‐tight H‐cell, rinsed with Milli‐Q water, and left to dry before its use.

### 4.9 Electrochemical Methods

#### 4.9.1 Electrochemical CO_2_ Reduction

The setup for electrochemical CO_2_ reduction consisted of a gas‐tight H‐cell with two compartments separated by a glass membrane (pore size 3 Å). The electrolyte solution employed was a 0.1 m KHCO_3_ aqueous solution (6.0 mL per compartment), prepared by dissolving 1.00 g of KHCO_3_ in Milli‐Q water and diluting to 100 mL. A conventional three‐electrode configuration was used for the CO_2_ electroreduction. The reference electrode (RE) consisted of an Ag/AgCl (3 m NaCl) electrode, the counter electrode (CE) a platinum wire, and the working electrode (WE) consisted of a drop‐cast Cu_2_O on a carbon paper. The WE and RE were placed in one compartment, and the CE electrode in the other. The WE was fixed by a crocodile connected to the copper tape stuck to the carbon paper. All metallic parts were coated with Teflon to prevent the electrolyte from contacting them. Once the cell was tightly sealed with septa, the compartments were purged with CO_2_ by bubbling the gas for at least 15 min, yielding a CO_2_‐saturated electrolyte with pH 6.8.

CO_2_ electroreduction was performed using an OrigaFlex 01A potentiostat by applying a fixed reductive potential for 1 h. Each experiment was performed using a new, unused electrode, and results are reported as the average of four independent measurements, with corresponding error bars.

Linear sweep voltammetry was performed at a scan rate of 100 mV s^−1^, ranging from −210 to −2200 mV vs Ag/AgCl or CE overload. Chronoamperometry (CA) was performed by applying the selected potential for 1 h. The overall charge that passed through the system can be obtained by integrating the current density (*j*) vs. time (*t*) curve. All reference potentials were converted to reversible hydrogen electrode (RHE) by using the following equation:

(1)

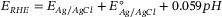

where *E°_Ag/AgCl_* = 0.197 V and *E_Ag/AgCl_* is the applied working potential. The geometric electrode surface area was normalized to all current densities.

#### 4.9.2 Potential Underpotential Deposition

The Pb underpotential deposition (Pb‐UPD) experiments were carried out on reduced Cu_2_O drop‐cast carbon paper electrodes in a pH 3 aqueous solution containing 0.1 M KClO_4_, 2 mM NaCl, 2 mM Pb(ClO_4_)_2_, and 1 mM HClO_4_. Cyclic voltammograms were recorded between –0.40 and –0.20 V vs Ag/AgCl at a scan rate of 25 mV s^−1^.

#### 4.9.3 CO_2_ Reduction Product Analysis

The CO_2_ reduction gas products were quantified using an Agilent 8890 gas chromatograph (GC) equipped with three columns connected in parallel (DB‐1, HP‐MOLESIEVE, and HP‐PLOT/Q) to ensure proper peak separation and resolution. A thermal conductivity detector (TCD) was used to measure hydrogen, while a methanizer combined with a flame ionization detector (FID) was used to measure carbon‐based products with higher sensitivity. When an electrochemical experiment was finished, 0.5 mL of the cathodic headspace (where the CO_2_ reduction takes place) was injected into the GC using a tight‐lock Hamilton syringe. To quantify the products, the GC was calibrated with three reference mixtures of H_2_, CO, CH_4_, and C_2_H_4_, and the volume of the cathodic headspace was measured beforehand.

The liquid products resulting from the CO_2_ electroreduction were quantified at Servei de Ressonància Magnètica Nuclear de la Universitat Autònoma de Barcelona by means of nuclear magnetic resonance (NMR) spectroscopy using a Bruker 300 MHz Ascent instrument with AVANCE NEO nanoconsole, AVAILABLE PROBE, and BCU‐05 complement for sample temperature control. Samples for analysis were prepared by mixing 650 µL of electrolyte with 50 µL of a 10 mM DMSO solution in D_2_O. DMSO was used as an internal pattern for the proper quantification of the products. All measurements were performed with water signal suppression to maximize the signals of DMSO and the products of interest. In all experiments, traces of 1‐propanol and acetic acid were detected, but their limited concentration prevented quantification.

#### 4.9.4 Faradaic Efficiency

Many reactions occur simultaneously, and the charge is distributed to form different products. Each product requires a given number of electrons to be formed, which is known (e.g., H_2_ 2e^−^, CO 2e^−^, HCOO^−^ 2e^−^, CH_4_ 8e^−^, C_2_H_4_ 12e^−^). This way, from the amount of generated product, Faradic efficiency can be calculated as follows:

(2) $$\begin{array}{c} \mathrm{Faradaic}\;\mathrm{efficiency}\;\;\left(\%\right)=\frac{Q_{prod}}{Q_{total}}\cdot 100=\frac{z_{prod}\cdot \;n_{prod}\cdot F}{Q_{total}}\cdot 100 \end{array}$$

where 𝑧_𝑝𝑟𝑜𝑑_ is the number of electrons required to reduce one molecule of CO_2_ (or H_2_O/H^+^ in the case of competitive hydrogen evolution reaction) to the specific product, 𝑛_𝑝𝑟𝑜𝑑_ are the moles of product generated during the electrolysis, and 𝐹 is the Faraday constant (96485 C mol^−1^).

#### 4.9.5 Calculation of Electrochemically Active Surface Area (ECSA)

The electrochemically active surface area (ECSA) of Cu_2_O_standard_, Cu_2_O_fluidic_, PAF‐coated Cu_2_O_standard,_ and PAF‐coated Cu_2_O_fluidic_ was computed by the double‐layer capacitance (C_dl_) inferred from applying a series of cyclic voltammetries (CV) at different scan rates (10‐500 mV s^−1^) in a non‐Faradaic region. The capacitive charging current (Equation 3) extracted at the midpoint potential was plotted as a function of the scan rate. The slope of the resulting linear regression line, forced through the origin, corresponds to twice the double‐layer capacitance. The ECSA was subsequently calculated using the relation (Equation 4), where C_s_ represents the specific capacitance of an atomically flat surface. As reported, the C_s_ depends on the nature of the material used and solution (for C, in neutral conditions C_s_ = 13–17 µF cm^−2^). The roughness factor (RF) (Equation 5) was determined by normalizing the calculated ECSA against the geometric surface area of the electrode (GSA).

(3) $$\Delta_{i}=\left|i_{a}-i_{c}\right|$$

(4) $$ECSA=\frac{C_{dl}}{C_{s}}$$

(5) $$RF=\frac{ECSA}{GSA}\;$$

#### 4.9.6 Flow Cell

A gas diffusion layer (Freudenberg H23C6) was acquired from Quintech. A suspension of Cu_2_O_fluidic_ in THF (2 mL, 1.5 mg mL^−1^) was drop‐cast onto a 4 cm^2^ gas diffusion layer. The PAF layer was generated in a one‐compartment cell containing an aqueous, CO_2_‐saturated (0.1 m KHCO_3_) solution of 10 mM diphenyliodonium triflate. To generate the PAFs, –0.90 V vs RHE was applied for 10 min. After that, the electrode was removed from the gas‐tight H‐cell, rinsed with Milli‐Q water, and left to dry before its use.

The setup consisted of an H‐type electrochemical cell (FlexCell‐PP, Gaskatel) with two compartments separated by a proton exchange membrane (PEM, Nafion 117). The CO_2_ flow rate on the back side of the cathode was fixed at 150 mL min^−1^ using a mass flow controller (Bronkhorst). The WE and RE, the GDE containing the Cu catalyst, and a hydrogen reference electrode (Hydroflex (M‐v01‐0071)) were placed in the cathodic compartment, while the CE (a Pt mesh) was placed in the anodic compartment. CO_2_ electroreduction was performed by applying a fixed potential of –1.25 V vs RHE, using a BioLogic (SP‐150e) potentiostat. The results given correspond to single measurements. Gaseous products were measured using an online gas chromatography system (990 Micro GC, Agilent) with the inlet directly connected to the flow cell. H_2_, CO, and CH_4_ were detected using a single channel with an MS5A SS column and a TCD detector. CO_2_ and multicarbon products (C_2_H_4,_ C_2_H_6_) were detected via a second channel equipped with a Poraplot U column and a TCD detector. Calibration of the GC was done using gas mixtures of known concentrations. Liquid products were collected in intervals of 20 min and quantified by means of proton nuclear magnetic resonance (^1^H‐NMR) spectroscopy using a Bruker 400 instrument. Samples for analysis were prepared by mixing 490 µL of electrolyte with 90 µL of deuterium oxide (D_2_O) and 20 µL of a 0.2% v/v DMSO aqueous solution as an internal standard. All measurements were performed with water signal suppression to maximize the signals of DMSO and the products of interest.

### 4.10 X‐ray Absorption Spectroscopy

Powder samples were prepared by diluting the corresponding Cu‐based material with cellulose to form a pellet (φ = 5 mm), then placing the pellet in the sample holder and sealing it with 30 µm Kapton tape. The electrodes were prepared by drop‐casting a 1 mg mL^−1^ suspension of the corresponding powder samples in THF onto a carbon cloth (CT Carbon Cloth with MPL W1S1011, Fuelcell) electrode. The Cu K‐edge was utilized to conduct measurements on Cu_2_O_fluidic_ and Cu_2_O_standard_, Cu(0), Cu_2_O and CuO in transmission mode. Prepared electrodes Cu_2_O_fluidic_/CC and Cu_2_O_standard_/CC for operando experiments were analyzed in fluorescent mode. Spectra were acquired at the beamline CLAESS (static Cu K‐edge XAS and EXAFS) at ALBA synchrotron with the collaboration of beamline scientist and ALBA staff. The incident energy was selected using a Si (311) double‐crystal monochromator. Incident flux was ca. 5×10^11^ ph/sec using a beam size of ca. 1000 µm × 1000 µm. The incident energy was calibrated by assigning the first inflection point of the Cu foil to 8979 eV. Fluorescence spectra were recorded using a Multichannel Silicon‐drift fluorescence detector. Final spectra were processed and normalized using the Athena program, included in the DEMETER package [54].

### 4.11 X‐ray Photoelectron Spectroscopy

Experiments were carried out in the ESFOSCAN at CCiTUB, an instrument based on the PHI VersaProbe 4 from Physical Electronics (ULVAC‐PHI). Measurements have been performed with a monochromatic, focused X‐ray source (Al Kα line at 1486.6 eV) calibrated using the 3d_5/2_ line of Ag with a full width at half maximum (FWHM) of 0.6 eV. The area analyzed was a circle with a diameter of 100 microns, and the resolution selected for the spectra was 224 eV Pass Energy and 0.8 eV step^−1^ for the general spectra, and 55 eV Pass Energy and 0.2 eV step^−1^ for the spectra of the selected elements. The analysis and fitting of the spectra were performed using Multipak version 9.9.2. A combination of a low‐energy electron gun (less than 10 eV) and a low‐energy Argon ion gun (less than 5 eV) was used to discharge the samples when necessary. All measurements were performed in an ultra‐high vacuum chamber at a pressure between 5·10^−10^ and 5·10^−9^ Torr.

### 4.12 Water Contact Angle Measurements

Water contact angles were measured at room temperature using the sessile drop method on an optical goniometer (Theta Lite, Biolin Scientific). For each measurement, a droplet of Milli‐Q water, 5 µL in volume, was deposited onto the sample surface using an automated dispenser. Samples consisted of as‐prepared and PAF‐coated Cu_2_O deposited on carbon paper. The droplet profile was recorded immediately upon deposition to capture the static equilibrium state. To ensure statistical reliability, the left‐ and right‐profile contact angles of each droplet were evaluated using OneAttension software. The results are reported as the average of four independent measurements.

### 4.13 Numerical Simulations

Numerical simulations of fluid flow and mass transport in the microfluidic device were performed using a computational fluid dynamics approach based on the finite‐volume method. The steady‐state Navier‐Stokes, continuity, and species transport equations for incompressible Newtonian fluids were solved using the SIMPLE algorithm with second‐order upwind discretization. The system was modeled using a 2D axisymmetric geometry, assuming fluid properties equivalent to those of water. To mimic the experiments, the reactant solutions were continuously introduced through the central or lateral inlets at various flow rates. A no‐slip condition was assumed at all walls of the microfluidic device, and zero gradient was assumed at the outlet. More details on numerical simulations, including mesh‐independence testing and validation, are provided in Section 2 of the Supporting Information.

## Author Contributions

**Anh Tuan Ngo**: investigation, writing – review and editing. **Júlia Mayans**: writing – review and editing, investigation. **João Pedro Vale**: investigation, writing – review and editing, writing – original draft. **Ona Falcó**: writing – review and editing, investigation. **Teresa Andreu**: investigation, supervision. **Gerard Martí**: investigation, writing – review and editing. **Francesca Peiró**: supervision, investigation, writing – review and editing, conceptualization, funding acquisition. **Sònia Estradé**: supervision, writing – review and editing, investigation. **Mohamed Amazian**: investigation. **Josep Puigmartí‐Luis**: conceptualization, investigation, writing – review and editing, supervision, funding acquisition, writing – original draft. **Jordi García‐Antón**: investigation, conceptualization, supervision, writing – review and editing, funding acquisition, writing – original draft. **Lluís Yedra**: investigation, writing – review and editing, supervision, conceptualization, funding acquisition. **Carlota Casas**: investigation, writing – original draft, writing – review and editing. **Roc Matheu**: conceptualization, investigation, funding acquisition, writing – review and editing, writing – original draft, supervision. **Xavier Sala**: conceptualization, investigation, writing – review and editing, funding acquisition, writing – original draft, supervision. **Marcos Gil‐Sepulcre**: investigation, writing – review and editing. **Tiago Sotto Mayor**: investigation, supervision, writing – review and editing, funding acquisition, writing – original draft.

## Funding

AGAUR (2023CLIMA00011, 2021SGR00242, 2021SGR 00270), AEI (RYC2021‐031578‐I, PID2022‐137777NA‐I00, PID2023‐146787OB‐I00, CEX2021‐001202‐M, PID2022‐138543NB, RYC2022‐037722‐I, RYC2024‐050678‐I), European Union's Horizon Europe Research and Innovation Program (GA no: 101047081), Portugal national funds (FCT/MECI: CEFT, UID/00532/2025, UID/PRR/00532/2025, and ALiCE, LA/P/0045/2020).

## Conflicts of Interest

The authors declare no conflicts of interest.

## Supporting information




**Supporting File 1**: advs76512‐sup‐0001‐SuppMat.docx.

**Supporting File 2**: advs76512‐sup‐0002‐VideoS1‐S2.zip.

## Data Availability

The data that support the findings of this study are available from the corresponding author upon reasonable request.
