# Supplementary material for: Continuous‐Flow Microfluidic Synthesis Enhances C2+ Selectivity for Cu2O Catalysts
Source: Adv Sci (Weinh). 2026 Jul 14;13(55):e76512. doi: 10.1002/advs.76512 (PMC13366368; doi:10.1002/advs.76512)
Supplement: Supplementary file 1 [file ADVS-13-e76512-s001.docx]

Supporting Information

Continuous-flow microfluidic synthesis enhances C_2+_ selectivity for Cu_2_O catalysts.

*Carlota Casas^1,2^, Anh Tuan Ngo^3^, João Pedro Vale^4^, Ona Falcó^5^, Gerard Martí^2^, Mohamed Amazian,^3^ Júlia Mayans^1^, Sònia Estradé^5^, Marcos Gil-Sepulcre^6^, Teresa Andreu,^3^ Francesca Peiró^5^, Tiago Sotto Mayor^4^, Lluís Yedra^5,^*, Jordi García-Antón^2^, Josep Puigmartí-Luis^3,7,^*, Xavier Sala^2,^*_,_ Roc Matheu^1,^**

**Table of contents**

[1.	Supplementary data	2](#_Toc234332989)

[2.	Numerical simulation	22](#_Toc234332990)

[2.1.	Fluid properties	22](#_Toc234332991)

[2.2.	Numerical methods	22](#_Toc234332992)

[2.3.	Geometry and boundary conditions	22](#_Toc234332993)

[2.4.	Reaction kinetics	23](#_Toc234332994)

[2.5.	Mesh independence testing and validation	24](#_Toc234332995)

[2.6.	Numerical simulation results	25](#_Toc234332996)

[3.	References	31](#_Toc234332997)

### 1. **Supplementary data**

**Figure S1.**  Comparison of reactants mixing in segmented- and continuous-flow microfluidic devices. As shown in this figure, in segmented-flow devices (**a**), mixing is due to chaotic advection, whereas in continuous-flow microfluidic devices (**b**), mixing of reactants is only accomplished through molecular diffusion. Note that the latter is a unique method for establishing an effective reaction–diffusion (RD) area, just like the ones present in nature. In the figure, the yellow and blue colors indicate reactants, the white color the pure solvent area, and in green is the reaction area. Figure reproduced with permission from ref ^1^

**Figure S2.** Technical illustrations of the microfluidic device used in our experiments: (**A**) front view, (**B**), side view, (**C**) top view, and (**D**) isometric view. All dimensions are in millimeters.

**Figure S3.** TEM images of Cu_2_O_fluidic_ that were quenched with different solvents, such as Mili-Q, ethanol, methanol and dimethyl sulfoxide (DMSO). Ethanol was chosen as a solvent because it showed the most homogeneous nanoparticles.

**Figure S4.** Powder X-ray diffraction (PXRD) patterns for Cu_2_O_fluidic_ and Cu_2_O_standard_.

**Figure S5.** (**A**) Normalized Cu K-edge X-ray absorption near-edge structure (XANES) spectra of Cu_2_O_standard_ and Cu_2_O_fluidic_, along with Cu(0), Cu_2_O, and CuO references. *k*^3^-weighted (**B**) and Fourier transform of *k*^3^-weighted (**C**) Cu Extended X-ray absorption fine structure (EXAFS) spectra of Cu_2_O_Standard_ and Cu_2_O_fluidic_ along with Cu(0), Cu_2_O, and CuO references. Experimental data are shown as solid and dotted lines, whereas the fitted curves are displayed as dashed lines. Experimental spectra were fitted for over a *k*-range of 2–12 Å^−1^.

**Figure S6.** High-angle annular dark-field scanning transmission electron microscopy (HAADF-STEM) image and corresponding fluorescence elemental maps of C (red), Cu (blue), and O (green) for Cu_2_O_fluidic_ (FRR = 1, TFR = 250 µL min^-1^).

**Figure S7.** High-resolution transmission electron microscopy (HRTEM) images and fast fourier transform (FFTs) for Cu_2_O_fluidic_. Indexation of the patterns used $Pn\bar{3}m$ Cu_2_O structure.

**Figure S8.** HRTEM images for Cu_2_O_standard_. Indexation of the patterns used $Pn\bar{3}m$ Cu_2_O structure.

**Figure S9.** HAADF-STEM images of (**A**–**B**) Cu_2_O_fluidic_ and (**C**–**D**) Cu_2_O_standard_.

**Figure S10.** 3D reconstruction of particle shapes through HAADF-STEM electron tomography for (**A–F**) Cu_2_O_fluidic_ (**G–L**) Cu_2_O_standard_. From left to right: (**A**, **G**) HAADF images, (**B**–**E**, **H**–**K**) slices through the reconstructed volume in the planes marked by the arrows, and (**F**–**L)** isosurface representations.

**Figure S11.** N_2_ adsorption/desorption results for (**A**) Cu_2_O_fluidic_ and (**B**) Cu_2_O_standard_.

**Figure S12.** TEM images for Cu_2_O_fluidic_ at the same FRR (FRR = 1) but different TFR. (**A**) TFR = 125 µL min^-1^, (**B**) TFR = 500 µL min^-1^_,_ (**C**) TFR = 750 µL min^-1^, and (**D**) TFR = 1000 µL min^-1^.

**Figure S13.** TEM images of Cu_2_O nanoparticles prepared with the 3D continuous-flow microfluidic device at different FRRs and at TFR = 250 μL min^-1^. FRR = flow rate ratio, TFR = total flow rate.

**Figure S14.** HAADF-STEM image and corresponding fluorescence elemental maps of C (red), O (green), Cu (blue), and N (yellow) for Cu_2_O_fluidic_ (FRR = 4; TFR = 250 µL min^-1^), showing the nitrogen layer resulting from the polymerized hydrazine.

**Figure S15.** X-ray Photoelectron Spectroscopy (XPS) survey spectrum for Cu_2_O_fluidic_ at FRR = 4 (blue) and FRR = 1 (red).

**Figure S16.** XPS spectrum in the Cu 2p region for Cu_2_O_fluidic_ at FRR = 4 (blue) and FRR = 1 (red).

**Figure S17.** XPS spectrum in the N 1s region for Cu_2_O_fluidic_ at FRR = 4 (blue) and FRR = 1 (red). The N peak at ~ 400 eV for Cu_2_O_fluidic_ (FRR = 4) is close to the detection limit.

**Figure S18.** Examples of (**A**) gas chromatograms of FID and TCD detectors for the determination of gaseous products after 1 h of applied potential and (**B**) NMR spectrum of the electrolyte for the determination of formic acid, ethanol, acetate, and 1-propanol using DMSO as internal standard. The results correspond to the gas and liquid products, respectively, generated by Cu_2_O_fluidic_ after 1 hour of chronoamperometry at **–**1.25 V vs RHE in a CO_2_-saturated 0.1 M KHCO_3_ aqueous solution.

**Figure S19.** Faradaic efficiencies (bars) and current densities (black dots) of (**A**) Cu_2_O_fluidic_ (FRR = 1 and TFR = 250 μL min^-1^) at –1.05, –1.25, –1.40 V vs RHE for 1 h and (**B**) Cu_2_O_fluidic_ at –1.25 V vs RHE at TFR = 250 μL min^-1^ and different FRR.

**Table S1.** CO_2_ electroreduction Faradaic efficiencies and current densities for Cu_2_O_fluidic_ and Cu_2_O_standard_.

| Products | As-prepared Cu_2_O_standard_ | As-prepared  Cu_2_O_fluidic_ | PAF-coated Cu_2_O_standard_ | PAF-coated Cu_2_O_fluidic_ |
| --- | --- | --- | --- | --- |
| H_2_ | 41(4) | 31(2) | 31(1) | 20(1) |
| CO | 6(1) | 15(1) | 9(2) | 16(3) |
| HCOO^-^ | 11(1) | 16(1) | 10(1) | 10(1) |
| CH_4_ | 12(1) | 14(1) | 8(1) | 9(2) |
| C_2_H_4_ | 10(1) | 17(1) | 21(1) | 26(1) |
| EtOH | 8(1) | 7(1) | 10(2) | 18(2) |
| 1-PrOH | Traces^a^ | Traces^a^ | Traces^a^ | Traces^a^ |
| CH_3_COOH | Traces^a^ | Traces^a^ | Traces^a^ | Traces^a^ |
| Geometric current density (mA cm^-2^) | –10(1) | –10(2) | –9.3(1) | -8(1) |
| ECSA-normalized current density (mA cm^-2^) | –16(2) | –53(11) | –36(3) | –124(16) |

^a^Traces of 1-PrOH and CH_3_COOH were detected by ^1^H-NMR, but their low concentration prevented quantification.

|  | | | | | |
| --- | --- | --- | --- | --- | --- |
| **Figure S20.** Faradaic efficiencies (bars) and current densities (black dots) of Cu_2_O_fluidic_ (FRR = 1 and same applied potential of –1.25 V vs RHE) at different TFR. | | | | | |
|  | | | | | |
| **Figure S21.** Pb UPD cyclic voltammetry on (**A**) Cu_2_O_fluidic_ and (**B**) Cu_2_O_standard_. Reference values were obtained from single-crystal electrodes^2,3^. Table S2 shows values of the experimental peaks. | | | | | |
| **Table S2.** Peak potential values in mV on Cu_2_O_fluidic_, Cu_2_O_standard._ | | | | |  |
|  | **Cu(310)** | **Cu(110)** | **Cu(111)** | **Defects** | |
| Cu_2_O_fluidic_ | NA | –355 | NA | –310 | |
| Cu_2_O_standard_ | –370 | NA | –325 | NA | |

**Figure S22.** Electrochemically active surface area (ECSA) of (**A**) as-prepared Cu_2_O_standard_, (**B**) PAF-coated Cu_2_O_standard_, (**C**) as-prepared Cu_2_O_fluidic_, and (**D**) PAF-coated Cu_2_O_fluidic_.

| **Table S3.** Electrochemically active surface area (ECSA) and roughness factor (RF) for as-prepared and PAF-coated Cu_2_O_standard_ and Cu_2_O_fluidic._ The geometric area was 0.311 cm^2^ for all electrodes. | | |
| --- | --- | --- |
|  | **ECSA (cm^2^)** | **RF** |
| As-prepared Cu_2_O_standard_ | 0.20 | 0.63 |
| As-prepared Cu_2_O_fluidic_ | 0.06 | 0.19 |
| PAF-coated Cu_2_O_standard_ | 0.08 | 0.25 |
| PAF-coated Cu_2_O_fluidic_ | 0.02 | 0.07 |

**Figure S23.** Normalized Cu K-edge XANES (**A**) spectra of Cu_2_O_standard_/CC before and during CO_2_ reduction catalysis, applying a CPE at –1.25 V vs RHE in 0.1 M KHCO_3_ buffer saturated with CO_2_, along with Cu(0), Cu_2_O, and CuO references. *k*^3^-weighted (**B**) and Fourier transform of *k*^3^-weighted (**C**) Cu EXAFS spectra of Cu_2_O_fluidic_/CC before and during CO_2_ reduction catalysis under the same conditions. Experimental data are shown as solid and dotted lines, whereas the fitted curves are displayed as dashed lines. Experimental spectra were fitted for over a *k*-range of 2–12 Å^−1^. CC = carbon cloth.

**Figure S24.** Normalized Cu K-edge XANES spectra of (**A**) Cu_2_O_fluidic_/CC and (**B**) Cu_2_O_standard_/CC during CO_2_ reduction catalysis, applying a CPE at –1.25 V vs RHE in 0.1 M KHCO_3_ buffer saturated with CO_2_, along with Cu(0) and Cu_2_O references. The solid black and pink lines correspond to the experimental and reconstructed spectra, respectively, obtained by linear combination fitting using Cu(0) and Cu_2_O references. CC = carbon cloth.

**Table S4.** EXAFS fitting parameters for Cu *K*-edges EXAFS region ($S_{0}^{2}$ fixed to 0.9).

| **Sample** | **Region** | **Species** | **Shell** | **CN** | **R,** Å | **σ^2^(10^-3^),** Å^2^ | **E_0_,** eV | **R-factor** | **Reduced** |
| --- | --- | --- | --- | --- | --- | --- | --- | --- | --- |
|  |  |  |  |  |  |  |  |  | **Chi-square** |
| Cu_2_O_standard_ |  |  |  |  |  |  |  |  |  |
| Cu K-edge | k = 2 – 12 Å^-1^ | Cu_2_O | Cu-O | 2 | 1.84 | 3.7 | 2.8 | 0.020 | 2933 |
|  | R = 1.0 – 3.3 Å |  | Cu-Cu | 12 | 3.02 | 23.2 |  |  |  |
|  |  |  | Cu-O | 6 | 3.45 | 29.9 |  |  |  |
| Cu_2_O_fluidic_ |  |  |  |  |  |  |  |  |  |
| Cu K-edge | k = 2 – 12 Å^-1^ | Cu_2_O | Cu-O | 2 | 1.84 | 4.1 | 2.6 | 0.024 | 3808 |
|  | R = 1.0 – 3.3 Å |  |  |  |  |  |  |  |  |
|  |  |  | Cu-Cu | 12 | 3.02 | 23.7 |  |  |  |
|  |  |  | Cu-O | 6 | 3.43 | 32.4 |  |  |  |
| Cu_2_O_fluidic_/CC | | | | | | | | | |
| Cu K-edge | k = 2 – 12 Å^-1^ | Cu_2_O | Cu-O | 2 | 1.84 | 3.9 | 2.4 | 0.028 | 166 |
|  | R = 1.0 – 3.3 Å |  | Cu-Cu | 12 | 3.02 | 22.0 |  |  |  |
|  |  |  | Cu-O | 6 | 3.42 | 28.5 |  |  |  |
| Cu_2_O_fluidic_/CC at –1.25 V vs RHE | | | | | | | | | |
| Cu K-edge | k = 2 – 12 Å^-1^ | Cu_2_O/Cu(0)  (66/34) | Cu-O | 1.3 | 1.85 | 4.7 | -1.0 | 0.017 | 55 |
|  | R = 1.2 – 5.2 Å |  |  |  |  |  |  |  |  |
|  |  |  | Cu-Cu | 7.9 | 3.01 | 20.5 |  |  |  |
|  |  |  | Cu-Cu | 4 | 2.56 | 9.9 |  |  |  |
|  |  |  | Cu-Cu | 2 | 3.63 | 9.4 |  |  |  |
|  |  |  | Cu-Cu | 8 | 4.49 | 19.1 |  |  |  |
|  |  |  | Cu-Cu | 4 | 4.96 | 8.4 |  |  |  |
| Cu_2_O_standard_/CC | | | |  |  |  |  |  |  |
| Cu K-edge | k = 2 – 12 Å^-1^ | Cu_2_O | Cu-O | 2 | 1.84 | 3.9 | 2.5 | 0.023 | 68 |
|  | R = 1.0 – 3.3 Å |  | Cu-Cu | 12 | 3.02 | 22.4 |  |  |  |
|  |  |  | Cu-O | 6 | 3.42 | 32.2 |  |  |  |
| Cu_2_O_standard_/CC at –1.25 V vs RHE | | | | | | | | | |
| Cu K-edge | k = 2 – 12 Å^-1^ | Cu_2_O/Cu(0)  (55/45) | Cu-O | 1.1 | 1.84 | 2.5 | -2.6 | 0.022 | 69 |
|  | R = 1.2 – 5.2 Å |  |  |  |  |  |  |  |  |
|  |  |  | Cu-Cu | 6.6 | 3.01 | 21.6 |  |  |  |
|  |  |  | Cu-Cu | 5.4 | 2.54 | 9.8 |  |  |  |
|  |  |  | Cu-Cu | 2.7 | 3.60 | 12.7 |  |  |  |
|  |  |  | Cu-Cu | 10.8 | 4.46 | 13.9 |  |  |  |
|  |  |  | Cu-Cu | 3.0 | 4.96 | 5.2 |  |  |  |
|  | | | | | | | | |  |
|  | | | | | | | | |  |
| **Figure S25.** In-situ HAADF-STEM images for Cu_2_O_fluidic_ (FRR = 1; TFR = 250 µL min^-1^) (**A**) before, (**B**) and after applying ~–1.25 V vs RHE for 300 seconds. | | | | | | | | |  |

**Figure S26.** In-situ electron energy loss spectroscopy (EELS) elemental maps of N (red), O (green), Si (dark blue), Cu (pink) and Pt (light blue) of a region of the working electrode with microfluidic nanoparticles (FRR = 1; TFR = 250 µL min^-1^) after catalysis.

**Figure S27.** In-situ HAADF-STEM images for Cu_2_O_standard_ (**A**) before, (**B**), and after applying ~ –1.25 V vs RHE for 300 seconds.

**Figure S28.** Faradaic efficiencies (bars) and current densities (black dots) of Cu_2_O_fluidic_ (FRR = 1 and TFR = 250 μL min^-1^), following sequential chronoamperometric measurements at 1 h, 2 h, and 3 h, at a potential of –1.25 V vs RHE.

**Figure S29**. Representative optical images of static Milli-Q water contact angles on different supports, showing both the left and right droplet profile angles: (**A**) pristine carbon paper, (**B**) PAF-coated Cu_2_O_standard_ on carbon paper, and (**C**) PAF-coated Cu_2_O_fluidic_ on carbon paper.

| **Table S5.** Static Milli-Q water contact angles on as-prepared and PAF-coated Cu_2_O deposited on carbon paper. | | |
| --- | --- | --- |
|  | **Contact angle (º)** | **Wetting state** |
| Pristine carbon paper | 130 (2) | Hydrophobic |
| As-prepared Cu_2_O_standard_ | Close to zero | Superhydrophilic |
| As-prepared Cu_2_O_fluidic_ | Close to zero | Superhydrophilic |
| PAF-coated Cu_2_O_standard_ | 109 (2) | Hydrophobic |
| PAF-coated Cu_2_O_fluidic_ | 128 (2) | Hydrophobic |

**Figure S30. (A**) Chronoamperometry and (**B**) Faradaic efficiencies for PAF-coated Cu_2_O_fluidic_ in a gas diffusion electrode incorporated in a flow cell at –1.25 V vs RHE.

**Table S6.** Comparison of different systems reported in literature about molecular and polymeric coatings on Cu.

| **Catalyst** | **Polymer / molecular coating** | **Cell type** | **Catholyte** | **E vs RHE (V)** | **C_2+_ FE without coating (%)** | | | **C_2+_ FE with coating (%)** | **Reference** |
| --- | --- | --- | --- | --- | --- | --- | --- | --- | --- |
| Polycrystalline Cu | Polypyrrole | H-cell | 0.1 M KHCO_3_ | -1.00 | | 1.4 | 1.6 | | ^4^ |
| Polycrystalline Cu | Polypyrrole-Polythiophene | H-cell | 0.1 M KHCO_3_ | -1.00 | | 1.4 | 7.0 | | ^4^ |
| Polycrystalline Cu | Polyaromatic film from diphenyliodonium (DPI) | 1-compartment cell^a^ | 0.1 M KHCO_3_ + 10 mM DPI^b^ | -1.05 | | 15 | 42 | | ^5^ |
| Polycrystalline Cu | 1,1′-di-p-tolyl-1,1′,4,4′-bipyridine (T-bipyridine) film | Flow cell | 0.1 M KHCO_3_ | -0.96 | | 7.5 | 46.1 | | ^6^ |
| Polycrystalline Cu | Polyaromatic film from diphenyliodonium (DPI) | Flow cell | 0.1 M KHCO_3_ | -1.05 | | 26.0 | 73.0 | | ^7^ |
| Polycrystalline Cu | Polyaromatic film from phenyldiazonium (PD) | Flow cell | 0.1 M KHCO_3_ | -1.05 | | 26.0 | 70.1 | | ^7^ |
| Polycrystalline Cu | Phenantroline-derived thin films | Flow cell | 0.1 M KHCO_3_ | -1.00 | | 17 | 47 | | ^8^ |
| Polycrystalline Cu | Phenantroline-derived thin films | Flow cell | 0.1 M KHCO_3_ + 10mM phenantroline-derived ligand** | -1.00 | | 17 | 76 | | ^8^ |
| Cu_2_O prepared via mixing-dominated microfluidics | None | Flow cell | 0.1 M KHCO_3_ | -1.10 | | 31.9 | NA | | ^9^ |
| Cu_2_O_fluidic_ prepared via continuous-flow microfluidics | Polyaromatic film from diphenyliodonium | H-cell | 0.1 M KHCO_3_ | -1.25 | | 24 | 44 | | This work |
| Cu_2_O_fluidic_ prepared via continuous-flow microfluidics | Polyaromatic film from diphenyliodonium | Flow cell | 0.1 M KHCO_3_ | -1.25 | | NA | 40 | | This work |

**^a^ In this setup the electrolyte is constantly flowing. ^b^ The additive is present in the electrolyte only on experiments concerning functionalized Cu electrodes.**

### 3. **Numerical simulation**

#### 3.1. **Fluid properties**

The copper acetate (12.5 mM) and hydrazine (12.5 mM) solutions are aqueous and dilute. Therefore, we assumed their density and viscosity were those of water (1000 kg m^-3^ and 1 mPa·s, respectively). Additionally, the diffusion coefficients of these species in the numerical simulations were chosen based on the literature (Table S7). The diffusion coefficient of Cu_2_O is not known and, thus, it was assumed to be 10^-10^ m^2^ s^-1^, a typical value for large molecules in water.

**Table S7.** Diffusion coefficient for each species in numerical simulations.

| Species | Diffusion coefficients | Source |
| --- | --- | --- |
| NH_2_NH_2_ | $8.33\times{10}^{-10} m^{2} s^{-1}$ | ^10^ |
| Cu^2+^ | $7.33\times{10}^{-10} m^{2}s^{-1}$ | ^11^ |
| Cu_2_O | $1\times{10}^{-10} m^{2}s^{-1}$ | assumed |

#### 3.2. **Numerical methods**

The flow and mass transport inside the microfluidic device were simulated using a computational fluid dynamics approach. The velocity, pressure, and concentration fields were calculated using the finite volume method by coupling the Navier-Stokes equation for incompressible Newtonian fluids, the continuity equation, and the species transport equation by

$$\frac{\partial\vec{V}}{\partial t}+\vec{V}\left( \nabla\cdot\vec{V} \right)=-\frac{1}{\rho}\nabla P+\upsilon\nabla^{2}\vec{V}$$

$$\frac{\partial\rho}{\partial t}+\nabla(\rho\vec{V})=0$$

$$\frac{\partial(\rho Y_{i})}{\partial t}+\nabla\left( \rho\vec{V}Y_{i} \right)={\rho D}_{i}\nabla^{2}Y_{i}+R$$

where $\vec{V}$ is the velocity vector, *P* is the pressure, ∇ is the divergence operator, $\nabla^{2}$ is the Laplacian operator, *ρ* is the fluid density, *υ* is the viscosity, *Y_i_* is the mass fraction of species i, *D_i_* is the diffusion coefficient of species i, and *R* is the rate of formation/consumption of i due to a chemical reaction. A steady-state solver was employed to solve these equations, using the SIMPLE algorithm for pressure-velocity coupling and second-order upwind discretization.

#### 3.3. **Geometry and boundary conditions**

Because the flow and mass transport in the main chamber of our microfluidic device are symmetric around the device's central axis (Figure S31a), we performed numerical simulations using an axisymmetric 2D geometry (Figure S31b), without loss of accuracy or representativeness of the results. To mimic the experiments, copper acetate and hydrazine solutions were continuously introduced through the central or lateral inlets at various flow rates. A no-slip condition was assumed at all walls of the microfluidic device, and a zero gradient was assumed at the outlet.

**Figure S31.** Representation of the simulation domain, main dimensions, symmetry axis and computational mesh.

#### 3.4. **Reaction kinetics**

The formation of Cu_2_O is a two-step reaction that involves the oxidation of hydrazine to release electrons, which are then responsible for the reduction of Cu^2+^. The two redox half-reactions and the overall reaction are represented in Figure S32.

**Figure S32.** Formation of Cu_2_O. The reaction is shown as the two redox half-reactions, and as the overall reaction.

There is little information in the literature on the kinetics of these reactions, nor could we find kinetic studies on similar reactions relevant to this process. Therefore, to simulate the formation of Cu_2_O, we focused on the overall reaction and ignored species that are likely less relevant to the process (e.g., acetate) and those that are abundant (e.g., water). This meant that we simulated the transport of Cu^2+^ and NH_2_NH_2_ and their reaction to form Cu_2_O in the microfluidic device:

$$4{Cu}^{2+}+{NH}_{2}{NH}_{2}+\ldots\to2{Cu}_{2}O+\ldots$$

With this simplified reaction in mind, we assumed that the formation of Cu_2_O followed a typical reaction rate law, given by:

$$r=k\left[ {Cu}^{2+} \right]\cdot[{NH}_{2}{NH}_{2}]$$

where *r* is the reaction rate, and *k* is the reaction rate constant. This reaction rate law implies that the rate exponents of Cu^2+^ and NH_2_NH_2_ are 1 and, therefore, that the reaction is of second order. Because the rate constant *k* is unknown, we ran simulations over a range of 1 to 10^6^ M^-1^∙s^-1^ and found that the results were qualitatively very similar. Therefore, we considered an intermediate value of k = 10^4^ M^-1^∙s^-1^ for all simulations. Because the reaction rate law considered here may not fully represent the actual reaction occurring in the device, we present the Cu_2_O concentration results as normalized relative to the highest concentration observed across all simulations.

#### 3.5. **Mesh independence testing and validation**

We performed preliminary simulations with different meshes to identify the coarsest that yielded mesh-independent results. These simulations were performed under typical experimental conditions, i.e., a total flow rate (TFR) of 250 µL min^-1^ and a flow rate ratio (FRR) of 1. We found that the velocity and Cu_2_O profiles along the device radius (Figure S33) obtained using a mesh containing 250 000 cells and convergence criteria of residuals lower than 10^-4^ were similar to those obtained with a mesh containing 1 250 000 cells and residuals lower than 10^-6^. Moreover, the velocity obtained with the 250 000 cell mesh and residuals lower than 10^-4^ was similar to the theoretical velocity solution for circular pipes.^12^ Therefore, we chose the mesh containing around 250 000 cells for all simulations (Figure S31c), and assumed that convergence was achieved when residuals were below 10^-4^.

**Figure S33**. Simulations for mesh independence testing and validation. (a) Velocity and (b) Cu_2_O concentration profiles along the radius at the outlet of the microfluidic device considering a TFR of 250 µL min^-1^ and an FRR of 1. The theoretical solution for velocity profile is valid for laminar flows in circular pipes.^12^ The finest mesh consists of 1 250 000 cells and its solution was considered converged when residuals were lower than 10^-6^. The chosen mesh consisted of 250 000 cells, and the solution was considered converged when the residuals were less than 10^-4^.

#### 3.6. **Numerical simulation results**

**Figure S34.** Concentration of Cu^2+^, NH_2_NH_2_ and Cu_2_O along the main microfluidic channel for TFR = 250 µL min^-1^ and FRR = 1.

**Figure S35.** Concentration of Cu^2+^, NH_2_NH_2_, and Cu_2_O at the (a) inlet and (b) outlet of the microfluidic device for TFR = 250 µL min^-1^ and FRR = 1.

**Figure S36.** Concentration of Cu^2+^, NH_2_NH_2_ and Cu_2_O along the main microfluidic channel for TFR = 125 µL min^-1^ and FRR = 1.

**Figure S37.** Concentration of Cu^2+^, NH_2_NH_2_ and Cu_2_O along the main microfluidic channel for TFR = 500 µL min^-1^ and FRR = 1.

**Figure S38.** Concentration of Cu^2+^, NH_2_NH_2_, and Cu_2_O along the main microfluidic channel for TFR = 750 µL min^-1^ and FRR = 1.

**Figure S39.** Concentration of Cu^2+^, NH_2_NH_2_ and Cu_2_O along the main microfluidic channel for TFR = 1000 µL min^-1^ and FRR = 1.

**Figure S40.** Numerical simulation results for constant FRR of 1 and TFR of 125, 500, 750 and 1000 µL min^-1^. (a) Cu_2_O outlet concentration profiles. (b) Maps of residence time (colour bar in log scale). (c) Maximum Cu_2_O concentration along the device. (d) Comparison between particle size and maximum Cu_2_O concentration.

**Figure S41.** Concentration of Cu^2+^, NH_2_NH_2,_ and Cu_2_O along the main microfluidic channel for TFR = 250 µL min^-1^ and FRR = 0.25.

**Figure S42.** Concentration of Cu^2+^, NH_2_NH_2_ and Cu_2_O along the main microfluidic channel for TFR = 250 µL min^-1^ and FRR = 4.

**Figure S43.** (a) Cu^2+^ concentration, (b) NH_2_NH_2_ concentration, (c) Cu_2_O concentration and (d) residence time at the outlet of the microfluidic channel for TFR = 250 µL min^-1^ and various FRR.

### 4. **References**

(1)	Puigmartí-Luis, J. Microfluidic Platforms: A Mainstream Technology for the Preparation of Crystals. *Chem. Soc. Rev.* **2014**, *43* (7), 2253–2271. https://doi.org/10.1039/C3CS60372E.

(2)	Nazari, P.; Zhao, S.; Christensen, O.; Sun, Z.; Ceccato, M.; Lauritsen, J. V.; Pedersen, S. U.; Rossmeisl, J.; Rosas-Hernández, A.; Daasbjerg, K. Enhancing Carbon Dioxide Reduction Performance on Copper via Surface Reconstruction Induced by Spontaneous Diazonium Salt Grafting. *J. Am. Chem. Soc.* **2025**, *147* (34), 31395–31408. https://doi.org/10.1021/jacs.5c11431.

(3)	Mazaira Couce, P.; Kongstad Madsen, T.; Plaza-Mayoral, E.; H. Kristoffersen, H.; Chorkendorff, I.; Nicole Dalby, K.; Stam, W. van der; Rossmeisl, J.; Escudero-Escribano, M.; Sebastián-Pascual, P. Tailoring the Facet Distribution on Copper with Chloride. *Chem. Sci.* **2024**, *15* (5), 1714–1725. https://doi.org/10.1039/D3SC05988J.

(4)	Venkatkarthick, R.; Lima, F. H. B. Polythiophene-Decorated Copper via Polypyrrole Intermediary Passivation Layer for Enhanced Electrocatalytic Reduction of Carbon Dioxide. *J. Electroanal. Chem.* **2024**, *961*, 118241. https://doi.org/10.1016/j.jelechem.2024.118241.

(5)	Vavra, J.; Ramona, G. P. L.; Dattila, F.; Kormányos, A.; Priamushko, T.; Albertini, P. P.; Loiudice, A.; Cherevko, S.; Lopéz, N.; Buonsanti, R. Solution-Based Cu+ Transient Species Mediate the Reconstruction of Copper Electrocatalysts for CO2 Reduction. *Nat. Catal.* **2024**, *7* (1), 89–97. https://doi.org/10.1038/s41929-023-01070-8.

(6)	Zhao, S.; Christensen, O.; Sun, Z.; Liang, H.; Bagger, A.; Torbensen, K.; Nazari, P.; Lauritsen, J. V.; Pedersen, S. U.; Rossmeisl, J.; Daasbjerg, K. Steering Carbon Dioxide Reduction toward C–C Coupling Using Copper Electrodes Modified with Porous Molecular Films. *Nat. Commun.* **2023**, *14* (1), 844. https://doi.org/10.1038/s41467-023-36530-z.

(7)	Watkins, N. B.; Lai, Y.; Schiffer, Z. J.; Canestraight, V. M.; Atwater, H. A.; Agapie, T.; Peters, J. C.; Gregoire, J. M. Electrode Surface Heating with Organic Films Improves CO_2_ Reduction Kinetics on Copper. *ACS Energy Lett.* **2024**, *9* (4), 1440–1445. https://doi.org/10.1021/acsenergylett.4c00204.

(8)	Nie, W.; Heim, G. P.; Watkins, N. B.; Agapie, T.; Peters, J. C. Organic Additive‐derived Films on Cu Electrodes Promote Electrochemical CO_2_ Reduction to C_2+_ Products Under Strongly Acidic Conditions. *Angew. Chem. Int. Ed.* **2023**, *62* (12), e202216102. https://doi.org/10.1002/anie.202216102.

(9)	Jun, M.; Kwak, C.; Lee, S. Y.; Joo, J.; Kim, J. M.; Im, D. J.; Cho, M. K.; Baik, H.; Hwang, Y. J.; Kim, H.; Lee, K. Microfluidics-Assisted Synthesis of Hierarchical Cu_2_O Nanocrystal as C_2_-Selective CO_2_ Reduction Electrocatalyst. *Small Methods* **2022**, *6* (5), 2200074. https://doi.org/10.1002/smtd.202200074.

(10)	Choudhry, K. I.; Svishchev, I. M.; Plugatyr, A. Split-Flow Taylor Dispersion Technique for Diffusivity and Concentration Measurements of Hydrazine in Aqueous Solution. *Can. J. Chem.* **2014**, *92* (4), 279–283. https://doi.org/10.1139/cjc-2013-0518.

(11)	PhreeqC (Version 3), 2025.

(12)	Bird, R. B.; Stewart, W. E.; Lightfoot, E. N. *Transport Phenomena*; John Wiley & Sons: New York, 1960.
